# β-Lactam Antibiotics Enhance the Pathogenicity of Methicillin-Resistant Staphylococcus aureus via SarA-Controlled Lipoprotein-Like Cluster Expression

**DOI:** 10.1128/mBio.00880-19

**Published:** 2019-06-11

**Authors:** Weilong Shang, Yifan Rao, Ying Zheng, Yi Yang, Qiwen Hu, Zhen Hu, Jizhen Yuan, Huagang Peng, Kun Xiong, Li Tan, Shu Li, Junmin Zhu, Ming Li, Xiaomei Hu, Xuhu Mao, Xiancai Rao

**Affiliations:** a Department of Microbiology, College of Basic Medical Sciences, Army Medical University (Third Military Medical University), Key Laboratory of Microbial Engineering under the Educational Committee in Chongqing, Chongqing, China; b Institute of Modern Biopharmaceuticals, School of Life Sciences, Southwest University, Chongqing, China; c Department of Clinical Microbiology and Immunology, College of Medical Laboratory Science, Army Medical University (Third Military Medical University), Chongqing, China; New York University School of Medicine

**Keywords:** β-lactam antibiotics, methicillin-resistant *Staphylococcus aureus*, SarA, TLR2, lipoprotein-like genes, pathogenicity

## Abstract

Methicillin-resistant Staphylococcus aureus (MRSA) resists nearly all β-lactam antibiotics that have a bactericidal activity. However, whether the empirically used β-lactams enhance MRSA pathogenicity *in vivo* remains unclear. In this study, we showed that a cluster of lipoprotein-like genes (*lpl*, *sa2275* to *sa2273* [*sa2275*–*sa2273*]) was upregulated in MRSA in response to subinhibitory concentrations of β-lactam induction. The increasing expression of *lpl* by β-lactams was directly controlled by the global regulator SarA. The β-lactam-induced Lpls stimulated the production of interleukin-6 and tumor necrosis factor alpha in RAW 264.7 macrophages. The *lpl* deletion mutants (N315Δ*lpl* and USA300Δ*lpl*) decreased the proinflammatory cytokine levels *in vitro* and *in vivo*. Purified lipidated SA2275-his proteins could trigger a Toll-like-receptor-2 (TLR2)-dependent immune response in primary mouse bone marrow-derived macrophages and C57BL/6 mice. The bacterial loads of N315Δ*lpl* in the mouse kidney were lower than those of the wild-type N315. The β-lactam-treated MRSA exacerbated cutaneous infections in both BALB/c and C57BL/6 mice, presenting increased lesion size; destroyed skin structure; and easily promoted abscess formation compared with those of the untreated MRSA. However, the size of abscesses caused by the β-lactam-treated N315 was negligibly different from those caused by the untreated N315Δ*lpl* in C57BL/6 TLR2^−/−^ mice. Our findings suggest that β-lactams must be used carefully because they might aggravate the outcome of MRSA infection compared to inaction in treatment.

## Abstract (precis)

β-Lactam antibiotics are widely applied to treat infectious diseases. However, certain poor disease outcomes caused by β-lactams remain poorly understood. In this study, we have identified a cluster of lipoprotein-like genes (*lpl*, *sa2275*–*sa2273*) that is upregulated in the major clinically prevalent MRSA clones in response to subinhibitory concentrations of β-lactam induction. The major highlight of this work is that β-lactams stimulate the expression of SarA, which directly binds to the *lpl* cluster promoter region and upregulates *lpl* expression in MRSA. Deletion of *lpl* significantly decreases proinflammatory cytokine levels *in vitro* and *in vivo*. The β-lactam-induced Lpls enhance host inflammatory responses by triggering the Toll-like-receptor-2-mediated expressions of interleukin-6 and tumor necrosis factor alpha. The β-lactam-induced Lpls are important virulence factors that enhance MRSA pathogenicity. These data elucidate that subinhibitory concentrations of β-lactams can exacerbate the outcomes of MRSA infection through induction of *lpl* controlled by the global regulator SarA.

## INTRODUCTION

Methicillin (MET)-resistant Staphylococcus aureus (MRSA) is a leading pathogen with notable pathogenic effects. MRSA causes a wide range of diseases, including acute skin and soft tissue infections, chronic and persistent endocarditis, osteomyelitis, and pneumonia (1, 2). MRSA infections cause higher morbidity and mortality than infections by MET-susceptible S. aureus (MSSA) (3, 4). However, the underlying mechanisms of these effects remain unclear. Studies have suggested that inappropriate treatments or unidentiﬁed virulence factors contribute to poor outcomes of MRSA infections (5, 6). Owing to failure to initially recognize MRSA infection, between 30% and 80% of individuals infected with MRSA have been reported to be inappropriately treated with β-lactam antibiotics (7, 8). Low levels of antibiotics can induce extracellular DNA release, biofilm formation, and virulence factor production (9, 10). Accumulated data have revealed that subinhibitory concentrations of β-lactam antibiotics can promote S. aureus pathogenicity by increasing the expression of alpha-toxin, Panton-Valentine leukocidin (PVL), enterotoxins, or staphylococcal protein A (SpA) *in vitro* (9–13). The contributions of certain altered virulence factors to MRSA pathogenicity *in vivo* and the molecular mechanisms underlying β-lactam-modulated MRSA pathogenicity remain largely unknown.

Over the past few decades, the global virulence regulon staphylococcal accessory (*sar*) and accessory gene regulators (*agr*) have been recognized to play central roles in S. aureus pathogenesis (14, 15). SarA is a pleiotropic global regulator that modulates the expression of approximately 120 genes in S. aureus via *agr*-dependent or -independent pathways (16). As a classic transcription factor, SarA can activate expressions of certain genes, for example, *agr* and *hla*, and repress expressions of others, such as *cna* and *sspA* (17). Treatment of S. aureus strains with subinhibitory concentrations of β-lactams showed increased SarA expression (12). However, whether β-lactam-induced SarA modulates other virulence factors to contribute to MRSA pathogenicity remains an important issue that must be addressed.

Lipoproteins (Lpps) are an abundant family of proteins anchored to the bacterial membrane and account for at least 2% of bacterial proteomes (18, 19). S. aureus encodes 55 to 70 putative Lpps, and approximately 50% of these Lpps are annotated as chaperones or transporters of amino acids, peptides, iron, and zinc (18). Several Lpps include major Toll-like receptor 2 (TLR2) ligands that play important roles in S. aureus infection and host inflammatory response (20). Several staphylococcal Lpps can trigger host cell invasion, increase bacterial pathogenicity, and contribute to the epidemic of CC8 and CC5 strains (21, 22). Other authors proposed that more than 30% of Lpps in S. aureus are hypothetically conserved proteins with unknown functions (19). Most virulent MRSA strains, such as USA300, carry a conserved genomic island termed νSaα, which is a nonphage and non-staphylococcal cassette chromosome genomic island that contains numerous homologous tandem-arranged *lpp* genes, which are referred to as “tandem Lpps” or “lipoprotein-like” (*lpl*) (18, 23, 24). This *lpl* cluster possibly represents the paralogous genes that have diverged after a duplication event in S. aureus (18). MRSA USA300, belonging to the clonal complex CC8, carries 15 (22%) hypothetical Lpls. Among these Lpls, nine are specific to the νSaα island (18). In contrast, N315, belonging to the clonal complex CC5, carries 12 (21%) hypothetical Lpls. Among these Lpls, nine Lpl proteins are specific to the νSaα island (locus 0) and three Lpls are encoded by the genome (locus III) (see Table S1 in the supplemental material) (24). However, the exact roles of Lpls remain unclear.

10.1128/mBio.00880-19.5 TABLE S1 Predicted Lpps of S. aureus N315. Download Table S1, DOCX file, 0.1 MB. Copyright © 2019 Shang et al. 2019 Shang et al. This content is distributed under the terms of the Creative Commons Attribution 4.0 International license.

In this study, we demonstrated that an *lpl* cluster outside the νSaα island was upregulated in response to subinhibitory concentrations of β-lactam induction. We observed that the increasing expression of *lpl* after β-lactam treatment was directly controlled by the global regulator SarA. We also showed that the β-lactam-induced Lpls are important virulence factors that enhance MRSA pathogenicity by triggering the TLR2-dependent expressions of interleukin-6 (IL-6) and tumor necrosis factor alpha (TNF-α).

## RESULTS

### β-Lactam antibiotics stimulated *lpl* expression in MRSA.

**FIG 1:**
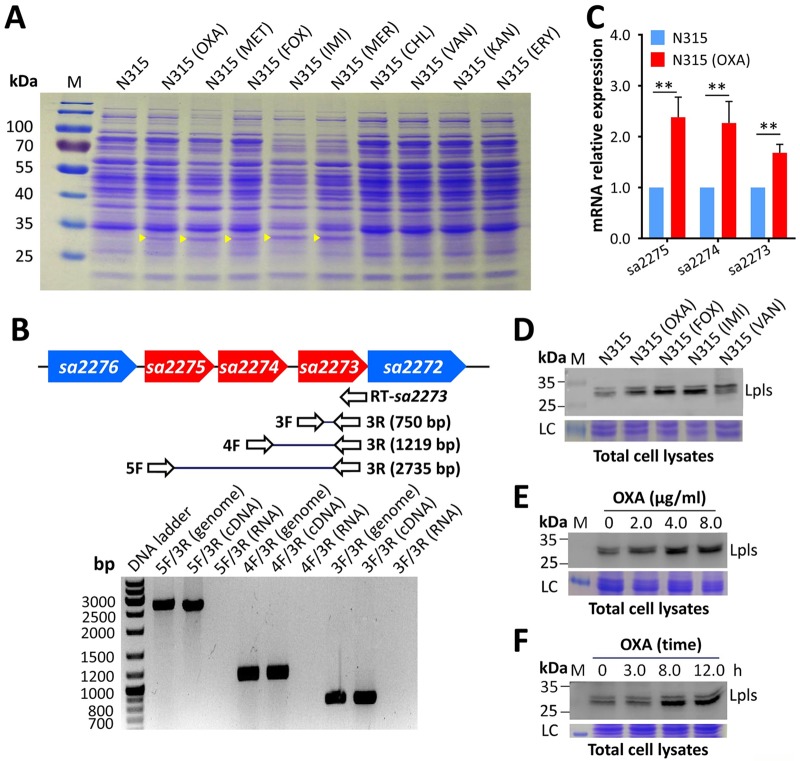
Upregulation of Lpls in MRSA posttreated with subinhibitory concentrations of antibiotics. (A) Proteins of MRSA N315 induced by different antibiotics were separated by SDS-PAGE and stained with Coomassie brilliant blue. An untreated N315 served as the negative control, and the molecular weights of the protein marker (M) are indicated on the left. The upregulated protein bands upon β-lactam antibiotic treatment are denoted by yellow triangles. (B) The *sa2275*, *sa2274*, and *sa2273* genes were cotranscribed as tested by RT-PCR. Genetic organization of *lpl* cluster in MRSA N315 genome and location of primers designed for RT-PCR. Agarose gel electrophoresis analysis of PCR products amplified with N315 genomic DNA, reverse-transcribed cDNA from N315 total RNA, and total RNA as the templates. The experiment was repeated three times, and a representative gel is shown. (C) RT-qPCR detection of the expression levels of *sa2275*, *sa2274*, and *sa2273* in N315 with or without OXA treatment was performed. The experiment was repeated three times. Error bars indicate standard deviation (SD). Statistical significance was calculated by Student’s *t* test. **, *P* < 0.01. (D) Western blot analysis of β-lactam-induced proteins in N315. (E) Western blot analysis of Lpl levels in N315 total cell lysates after OXA treatment at different concentrations. (F) Western blot analysis of Lpl levels in N315 after OXA treatment with increasing time. Western blot experiments were conducted using antibodies against SA2275-his (-sp) and repeated three times, and representative gels are shown. Molecular weights of the protein markers (M) are indicated on the left. LC, loading control.

β-Lactam antibiotics block the cell wall synthesis of bacteria to exert antimicrobial effects. In contrast, subinhibitory concentrations of β-lactam antibiotics are reported to induce the production of S. aureus toxins (12). A globally prevalent sequence type 5 (ST5) MRSA strain N315 (25) was tested for its antibiotic response to identify new factors contributing to MRSA pathogenicity. The MICs of oxacillin (OXA), MET, cefoxitin (FOX), imipenem (IMI), meropenem (MER), chloramphenicol (CHL), vancomycin (VAN), kanamycin (KAN), and erythromycin (ERY) against N315 were determined (see Table S2 in the supplemental material). Sodium dodecyl sulfate-polyacrylamide gel electrophoresis (SDS-PAGE) revealed that a protein band of approximately 30 kDa was upregulated in subinhibitory concentrations of OXA-, MET-, FOX-, IMI-, or MER-treated N315 compared with the untreated control (Fig. 1A). In contrast, CHL, VAN, KAN, and ERY showed no induction effects on this protein band. Further observations indicated that subinhibitory concentrations of OXA exerted a broad-spectrum induction effect on other major clinically prevalent MRSA clones (see Fig. S1A in the supplemental material).

10.1128/mBio.00880-19.1 FIG S1 Distribution and Western blot analysis of the β-lactam-induced Lpls. (A) Major clinically prevalent MRSA strains were cultured in the absence or presence of 2 μg/ml of OXA. SDS-PAGE was then performed. The yellow triangles indicate certain upregulated protein bands after OXA treatment. (B) Alignment of the SA2275, SA2274, and SA2273 signal peptides and lipobox sequences. (C) Alignment of SA2275, SA2274, and SA2273 proteins. The amino acid sequences of the indicated proteins were retrieved from UniProt (http://www.uniprot.org/), and the alignment was conducted using the BioEdit program. The identical amino acids were colored. (D) Lpl levels in N315 culture supernatant (CS) increased after treatment with different concentrations of OXA. (E) The expression of Lpls upregulated in N315 after treatment with different concentrations of MET. SarA proteins were also increased after MET treatment. The molecular weights of the protein markers (M) are indicated on the left. LC, loading control. Download FIG S1, TIF file, 2.0 MB. Copyright © 2019 Shang et al. 2019 Shang et al. This content is distributed under the terms of the Creative Commons Attribution 4.0 International license.

10.1128/mBio.00880-19.6 TABLE S2 MICs of MRSA strains. Download Table S2, DOCX file, 0.02 MB. Copyright © 2019 Shang et al. 2019 Shang et al. This content is distributed under the terms of the Creative Commons Attribution 4.0 International license.

The protein band was excised from the SDS-PAGE gel and analyzed through liquid chromatography tandem mass spectrometry (LC-MS/MS) to characterize the β-lactam-induced proteins in MRSA strains. The detected peptides matched with 68 proteins in the N315 proteome (see Table S3 in the supplemental material). Most known metabolic enzymes were excluded, and three putative Lpls, SA2273 (30.8 kDa), SA2274 (30.1 kDa), and SA2275 (30.4 kDa), which are encoded by a consecutive gene cluster, were selected on the basis of theoretical molecular weights for analysis (Fig. 1B; see Table S1). SA2273, SA2274, and SA2275 were annotated as Lpls in the N315 genome (locus III) (GenBank accession no. BA000018.3) (24). A typical Lpp precursor contains a signal peptide at the N-terminal end, and a characteristic conserved three-amino-acid lipobox is detected in front of the invariable cysteine [(LVI) (ASTG) (GA)↓**C**] (19, 20). Both SA2275 and SA2273 possess signal peptides and “lipobox” sequences, whereas SA2274 comprises a transmembrane helix domain at the N-terminal end (see Fig. S1B in the supplemental material). These proteins were annotated as Lpls that belong to a domain of unknown function 576 (DUF576) protein family on the Pfam database (26) and account for more than 62.6% of amino acid identity (see Fig. S1C in the supplemental material). To verify whether *sa2275*, *sa2274*, and *sa2273* are cotranscribed, reverse transcription-PCR (RT-PCR) of *sa2273* was performed with RNA extracted from wild-type N315 by specific primers. Results on the template of genomic DNA or RNA revealed that *sa2275*, *sa2274*, and *sa2273* were cotranscribed from the *sa2275* promoter (Fig. 1B). We further examined the influence of β-lactams on *lpl* expression. Quantitative RT-PCR (RT-qPCR) showed that the mRNA levels of *sa2275*, *sa2274*, and *sa2273* were upregulated in N315 after treatment with subinhibitory concentrations of OXA (Fig. 1C). The identities of β-lactam-induced proteins were verified through Western blot analysis (Fig. 1D). Two protein bands were detected by mouse anti-SA2275 antibody in Western blot experiments, and this result was probably due to the high identities of Lpls at the amino acid level. Western blot analysis also demonstrated that the protein levels of Lpls in N315 total cell lysates (Fig. 1E) and culture supernatant (see Fig. S1D in the supplemental material) increased in a dose-dependent manner after OXA treatment. Lpl expression was also upregulated in N315 after MET treatment (see Fig. S1E in the supplemental material). Furthermore, the Lpl expression by N315 increased in a time-dependent manner after OXA treatment (Fig. 1F). These results verified that MRSA Lpls could be released from bacteria, and their production was influenced by subinhibitory concentrations of β-lactam antibiotics.

10.1128/mBio.00880-19.7 TABLE S3 Proteins identified in the protein band of β-lactam-induced MRSA N315 by LC-MS/MS. Download Table S3, DOCX file, 0.04 MB. Copyright © 2019 Shang et al. 2019 Shang et al. This content is distributed under the terms of the Creative Commons Attribution 4.0 International license.

### β-Lactam-induced Lpl expression in MRSA was directly controlled by SarA.

**FIG 2:**
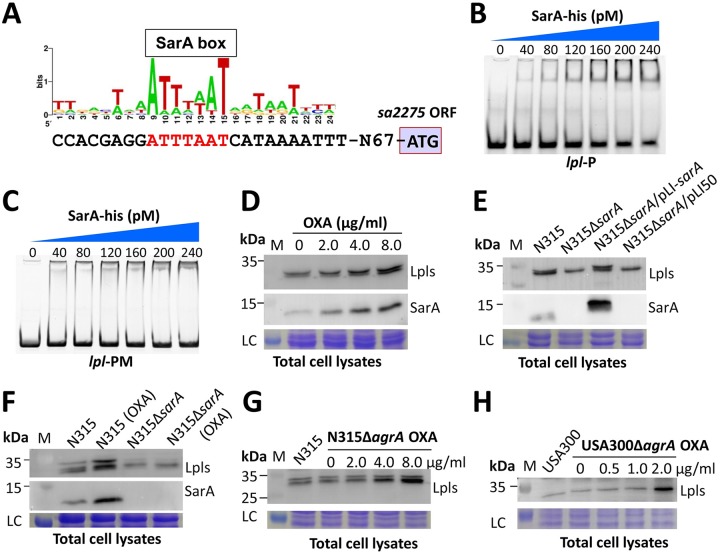
SarA bound to the *lpl* cluster promoter region to control β-lactam-induced Lpl expression in MRSA. (A) Predicted SarA box in the promoter regions of the *lpl* cluster. (B) EMSA detected the interaction between wild-type *lpl* promoter region (*lpl*-P) and SarA-his proteins. (C) EMSA by *lpl* promoter mutant (*lpl*-PM). (D) Western blot analysis of SarA and Lpls in N315 treated with different concentrations of OXA. (E) Deletion of *sarA* decreased Lpl expression in N315. (F) Western blot analysis of SarA and Lpls in N315 and N315Δ*sarA* postcultured in BHI with or without OXA treatment. (G) Lpl levels in N315Δ*agrA* increased after treatment with different concentrations of OXA. (H) Lpl levels in USA300Δ*agrA* increased after treatment with different concentrations of OXA. Western blot assays and EMSAs were repeated three times, and representative gels are shown. Molecular weights of the protein marker (M) are indicated on the left. LC, loading control.

As inducers, β-lactams may trigger global regulatory networks to modulate virulence in S. aureus; the global virulence regulons *sar* and *agr* play critical roles in virulence factor production (12, 14). Global regulators can recognize specific motifs in the promoter regions of a certain gene, thereby controlling gene expression (14). We analyzed the binding motif of SarA (27, 28) in the promoter regions of *lpl* and discovered a typically predicted SarA box (Fig. 2A). Electrophoretic mobility shift assay (EMSA) results showed that recombinant SarA-his proteins bound to the *lpl* cluster promoter region that carried the putative SarA binding box (Fig. 2B). No shifting band was observed when the AT-rich SarA box was mutated to become GC rich (Fig. 2C). To investigate whether β-lactam-stimulated *sarA* can regulate the expression of *lpl*. Western blot analysis indicated that both SarA and Lpls increased in a dose-dependent manner in response to β-lactam antibiotic treatment (Fig. 2D). Deletion of *sarA* (N315Δ*sarA*) reduced Lpl levels in N315 (Fig. 2E). The *sarA-*overexpressing strain (N315Δ*sarA*/pLI-*sarA*) produced more Lpls than the wild-type N315 strain, whereas the empty pLI50-transformed strain (N315Δ*sarA*/pLI50) caused no such effect. The Lpls presented no significant change in the *sarA* mutant after OXA treatment compared with untreated N315Δ*sarA* (Fig. 2F). However, OXA treatment significantly increased the Lpl levels in N315. These data indicate that S. aureus SarA can directly bind to the *lpl* cluster promoter region, thereby upregulating *lpl* expression in the presence of β-lactams. The AT-rich motif (ATTTAAT) in the promoter regions of *lpl* is essential for SarA binding and regulation.

We then examined whether AgrA can upregulate the expression of *lpl*. Western blot analysis showed that N315 and N315Δ*agrA* produced similar amounts of Lpls, and Lpl levels in N315Δ*agrA* increased after OXA treatment (Fig. 2G). Studies have reported that N315 contains a defective *agr* (29). We then tested the effect of *agr* on *lpl* expression in an *agr*-positive MRSA USA300 strain. USA300Δ*agrA* expressed similar amounts of Lpls as the wild-type strain, and Lpl levels in USA300Δ*agrA* also increased after OXA treatment (Fig. 2H). These data indicate that MRSA *agr* plays no role in the regulation of Lpl expression. The β-lactam-induced Lpl expression in MRSA may be controlled by SarA through an *agr*-independent pathway.

### β-Lactam-induced Lpls triggered TLR2-dependent proinflammatory cytokine production by macrophages.

**FIG 3:**
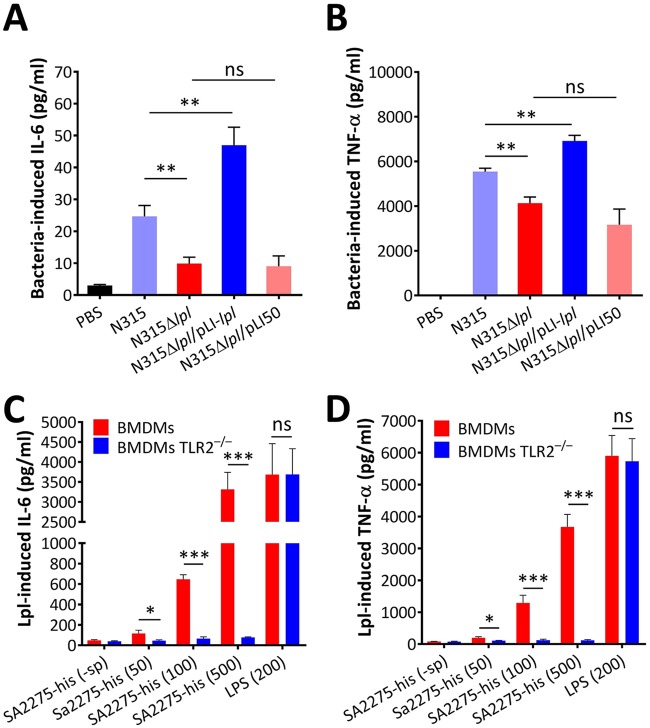
Antibiotic-induced MRSA Lpls stimulated proinflammatory cytokine production by macrophages. IL-6 (A) and TNF-α (B) levels elevated by RAW 264.7 macrophages stimulated with N315, N315Δ*lpl*, N315Δ*lpl*/pLI-*lpl*, or N315Δ*lpl*/pLI50 at the MOI of 30. The levels of IL-6 and TNF-α in cell culture supernatant were determined 6 h postinfection through enzyme-linked immunosorbent assay (ELISA). Phosphate-buffered saline (PBS) served as negative controls. Approximately 5 × 10^5^ BMDMs or BMDM TLR2^−/−^ cells were stimulated with different amounts of purified lipidated SA2275-his proteins (50, 100, and 500 ng/ml). IL-6 (C) and TNF-α (D) levels in cell culture supernatant were determined after 6 h of treatment. The unlipidated SA2275-his (-sp) (500 ng/ml)-stimulated cells served as the negative control, whereas LPS-induced cells (200 ng/ml) served as the positive control. The experiments in duplicate were conducted at least three times. Error bars indicate SD. Statistical significances were calculated by Student’s *t* test; ns, no statistical significance. *, *P* < 0.05; **, *P* < 0.01; ***, *P* < 0.001.

The νSaα-specific Lpl proteins of MRSA USA300 enhanced the production of IL-6 and TNF-α in innate immune cells (21). In N315, the β-lactam-inducible *lpl* genes are not in the νSaα island and encode Lpls belonging to the DUF576 protein family (24, 26). To determine whether Lpls contribute to innate immune stimulation, we constructed a markerless deletion mutant, N315Δ*lpl*, and a complement strain, N315Δ*lpl*/pLI-*lpl*, for macrophage infection (see Fig. S2A in the supplemental material). The production of IL-6 and TNF-α in mouse RAW 264.7 macrophages significantly decreased after treatment with N315Δ*lpl* compared with those of wild-type N315 administered. In contrast, higher levels of IL-6 and TNF-α were detected in macrophages treated with N315Δ*lpl*/pLI-*lpl* than with N315Δ*lpl*/pLI50 (Fig. 3A and B). This effect was also observed in RAW 264.7 macrophages stimulated with MRSA USA300, USA300Δ*lpl*, USA300Δ*lpl*/pLI-*lpl*, and USA300Δ*lpl*/pLI50 (see Fig. S2B, C, and D in the supplemental material). These results indicate that the increased levels of IL-6 and TNF-α by macrophages may depend on the expression of MRSA Lpls.

10.1128/mBio.00880-19.2 FIG S2 Effect of MRSA USA300 Lpls on the stimulation of proinflammatory cytokines by macrophages. (A) Schematics for construction of the *lpl* markerless deletion plasmid pYT3-Δ*lpl* and the complementation plasmid pLI-*lpl*. (B) Western blot analysis of Lpls in USA300, USA300Δ*lpl*, USA300Δ*lpl*/pLI-*lpl*, and USA300Δ*lpl*/pLI50 strains. Results are representative gels from three independent experiments. Molecular weights of the protein markers (M) are indicated on the left. LC, loading control. (C and D) IL-6 (C) and TNF-α (D) levels elevated by RAW 264.7 macrophages stimulated with MRSA USA300, USA300Δ*lpl*, USA300Δ*lpl*/pLI-*lpl*, or USA300Δ*lpl*/pLI50 at the MOI of 30. The experiments in duplicate were conducted at least three times. Error bars indicate SD. Statistical significances were calculated by Student’s *t* test; ns, no statistical significance. *, *P < *0.05. Download FIG S2, TIF file, 0.4 MB. Copyright © 2019 Shang et al. 2019 Shang et al. This content is distributed under the terms of the Creative Commons Attribution 4.0 International license.

In Gram-negative bacteria, the cell-wall-associated lipopolysaccharides (LPSs) are the main molecules involved in activating the innate immune system of hosts via TLR4 interaction (30), whereas in Gram-positive bacteria, the releasable Lpps or Lpls may be the main factors, performing a similar function by triggering the TLR2 signaling pathway and thereby inducing proinflammatory cytokine production (20). To determine whether β-lactam-induced Lpls exhibit immunomodulatory effects on primary macrophages, mouse bone marrow-derived macrophages (BMDMs) of C57BL/6 mice were isolated and characterized and then stimulated with different amounts of purified lipidated SA2275-his proteins. SA2275-his proteins increased the production of IL-6 and TNF-α in BMDMs in a concentration-dependent manner, whereas unlipidated SA2275-his (-sp) proteins showed no immunomodulatory effect on the production of IL-6 and TNF-α in BMDMs (Fig. 3C and D). However, the lipidated SA2275-his proteins could not induce the production of IL-6 and TNF-α in BMDMs derived from TLR2-deficient C57BL/6 mice (TLR2^−/−^ BMDMs) (Fig. 3C and D). In summary, these data suggest that the enhancement by purified recombinant SA2275-his proteins of proinflammatory cytokine production by macrophages is TLR2 dependent, and correctly lipidated Lpls are needed for the recognition of TLR2 receptors to trigger the immune response by macrophages (31).

### MRSA Lpls enhanced proinflammatory cytokine production in mice.

**FIG 4:**
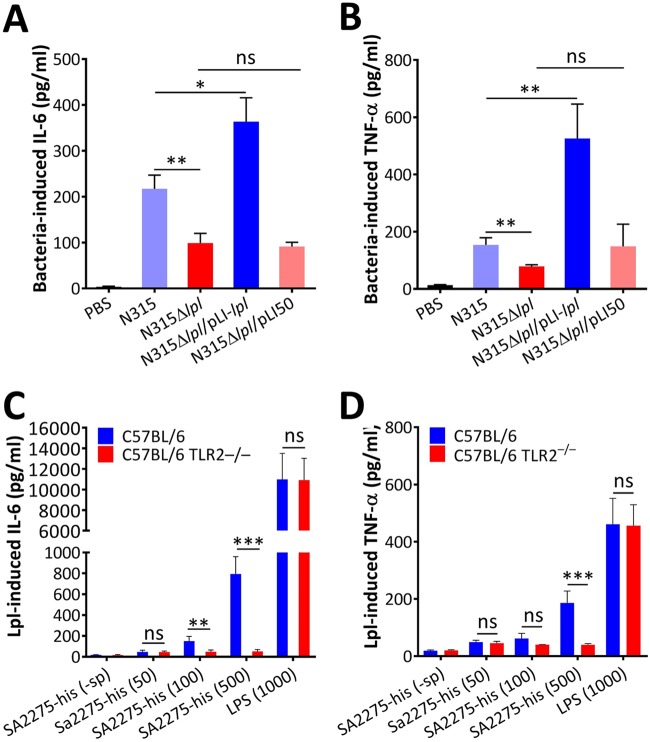
Lpls contributed to inflammatory response in MRSA infection. IL-6 (A) and TNF-α (B) levels in BALB/c mouse sera as determined by ELISA. BALB/c mice were infected by tail vein injection with 1 × 10^7^ CFU of N315, N315Δ*lpl*, N315Δ*lpl*/pLI-*lpl*, or N315Δ*lpl*/pIL50. The levels of IL-6 and TNF-α in mouse sera were determined 6 h postinfection. PBS served as negative controls. C57BL/6 and C57BL/6 TLR2^−/−^ mice were injected in the tail vein with 50, 100, and 500 ng of purified lipidated SA2275-his proteins. IL-6 (C) and TNF-α (D) levels in mouse sera were determined 6 h posttreatment. Mice administered LPS (1,000 ng) and unlipidated SA2275-his (-sp) proteins (500 ng) served as the positive and negative controls, respectively. Data show mean ± SD for cytokine levels from three independent experiments with five mice in each group. Statistical significances were calculated by Student’s *t* test; ns, no statistical significance. *, *P* < 0.05; **, *P* < 0.01; ***, *P* < 0.001.

We determined whether the β-lactam responsible for Lpls contributed to cytokine stimulation *in vivo*. The levels of IL-6 and TNF-α in BALB/c mice 6 h after challenge with N315Δ*lpl* were significantly lower than those with N315 administered (*P* < 0.01). The N315Δ*lpl*/pLI-*lpl* strain stimulated higher levels of IL-6 and TNF-α in mice than the N315Δ*lpl*/pLI50 strain did (Fig. 4A and B). Moreover, C57BL/6 and C57BL/6 TLR2^−/−^ mice were administered different concentrations of purified lipidated SA2275-his proteins through tail vein injection. The levels of IL-6 and TNF-α in C57BL/6 mice increased after SA2275-his proteins were administered, whereas this phenomenon was absent in C57BL/6 TLR2^−/−^ mice (Fig. 4C and D). Overall, these data suggest that the systemic inflammatory response in MRSA infection may be associated with Lpl expression and that MRSA Lpls stimulate a TLR2-dependent host immune response.

### β-Lactam-induced Lpls promoted the pathogenicity of MRSA.

**FIG 5:**
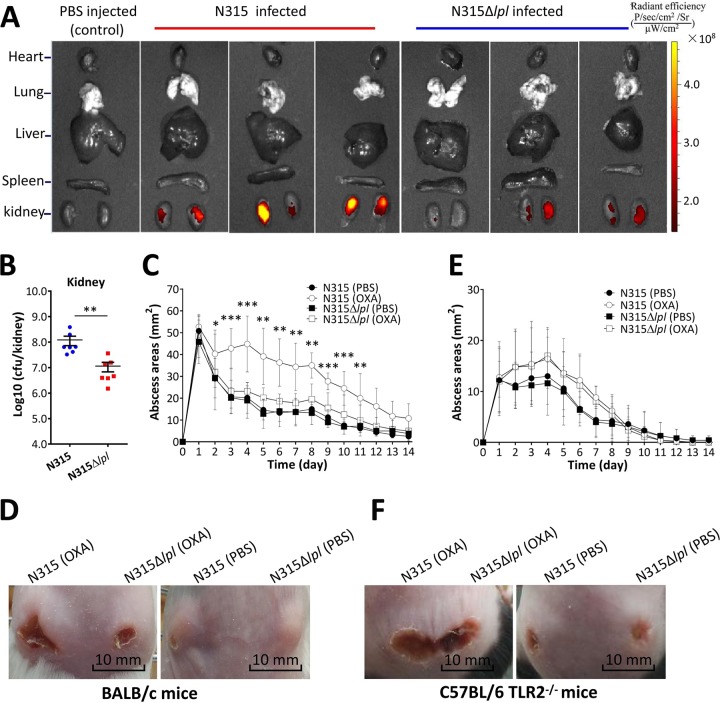
β-Lactam treatment enhanced the pathogenicity of MRSA in the mouse infection model. (A) Organ distribution of N315 and N315Δ*lpl.* BALB/c mice were infected with 1 × 10^7^ CFU of pGFP plasmid-transformed N315 and N315Δ*lpl*, and radiant efficiency of the indicated organs was measured with the IVIS Lumina LT system. (B) Bacterial loads in kidneys. BALB/c mice (*n *=* *7) were infected with 1 × 10^7^ CFU of N315 and N315Δ*lpl* for 5 days, and bacteria were recovered and counted from the kidneys. Data were analyzed using Mann-Whitney U test. **, *P* < 0.01. (C) BALB/c mice (*n *=* *5) were infected subcutaneously with 5 × 10^7^ bacterial cells and intraperitoneally treated with 1 μg of OXA per gram of weight twice a day for 14 days. Abscess areas were measured daily. Error bars indicate SD. Statistical significances were calculated by multiple *t* test. *, *P* < 0.05; **, *P* < 0.01; ***, *P* < 0.001. (D) Representative abscesses at 7 days after infection. (E) C57BL/6 TLR2^−/−^ mice (*n *=* *5) were injected subcutaneously with 5 × 10^7^ bacterial cells and intraperitoneally treated with 1 μg of OXA per gram of weight twice a day for 14 days. Abscess areas were measured daily. Error bars indicate SD. Statistical significances were calculated by multiple *t* test, and no statistical significance was observed. (F) Representative abscesses of C57BL/6 TLR2^−/−^ mice at 7 days after infection.

To investigate whether β-lactam-induced Lpls enhance colonization by MRSA, we determined the bacterial burden in the organs of a mouse model. BALB/c mice were infected intravenously with pGFP plasmid-transformed N315Δ*lpl* and N315 for 5 days (see Table S4 in the supplemental material), and bacterial colonization was tracked through an *in vivo* imaging system. The fluorescence intensity of green fluorescent protein (GFP) in the kidneys of mice injected with N315 was significantly higher than in those infected with N315Δ*lpl* (Fig. 5A; see Fig. S3A in the supplemental material). Consistent with the radiant efficiency, the bacterial loads in the kidneys of N315-infected mice were also significantly higher than those of N315Δ*lpl-*infected ones (Fig. 5B).

10.1128/mBio.00880-19.3 FIG S3 β-Lactam-induced Lpls contribute to the pathogenicity of MRSA in the BALB/c mouse infection model. (A) Lpls contributed to the colonization by MRSA. The radiant efficiency of N315-infected organs was higher than that of N315Δ*lpl*-infected ones. Data show mean ± SD for three mice in each group from two independent experiments. Statistical significances were calculated by Student’s *t* test. *, *P < *0.05. (B) BALB/c mice were subcutaneously injected in both flanks with 5 × 10^7^ CFU of N315 and N315Δ*lpl* cells and intraperitoneally injected with 1 μg of OXA per gram of weight twice a day for 7 days. PBS-treated N315- and N315Δ*lpl*-challenged mice served as controls. The skin abscesses caused by the OXA-treated N315 were larger than those caused by the OXA-treated N315Δ*lpl*, PBS-treated N315, and PBS-treated N315Δ*lpl*. (C) Representative histological examinations (H&E stain) of the infected mouse skin. (D and E) IL-6 (D) and TNF-α (E) levels in mouse sera determined by ELISA. BALB/c mice were infected by tail vein injection with 1 × 10^7^ CFU of N315, OXA-treated N315, or OXA-treated N315Δ*lpl*. The levels of IL-6 and TNF-α in mouse sera were determined 6 h postinfection. PBS and OXA served as controls. Experiments in duplicate were conducted at least three times. Error bars indicate SD. Statistical significances were calculated by Student’s *t* test; ns, no statistical significance. *, *P < *0.05; **, *P < *0.01. Download FIG S3, TIF file, 1.8 MB. Copyright © 2019 Shang et al. 2019 Shang et al. This content is distributed under the terms of the Creative Commons Attribution 4.0 International license.

10.1128/mBio.00880-19.8 TABLE S4 Strains and plasmids used in this study. Download Table S4, DOCX file, 0.03 MB. Copyright © 2019 Shang et al. 2019 Shang et al. This content is distributed under the terms of the Creative Commons Attribution 4.0 International license.

We then investigated whether Lpls enhance the pathogenicity of MRSA. A mouse subcutaneous infection model was established to evaluate the contribution of OXA-induced Lpls to skin and soft tissue infections. BALB/c mice were injected subcutaneously in both flanks with N315 and N315Δ*lpl*. Then, the mice were intraperitoneally injected with 1 μg of OXA per gram of weight twice a day for 14 days. The course of infection was monitored every day. The PBS-treated N315- and N315Δ*lpl*-infected mice served as controls. The mouse skin abscesses caused by OXA-treated N315 were significantly larger than those caused by OXA-treated N315Δ*lpl*, PBS-treated N315, and PBS-treated N315Δ*lpl* between 2 and 11 days postinfection (Fig. 5C), and this observation was further shown in the photographs of skin abscesses of BALB/c mice 7 days postinfection (Fig. 5D; see Fig. S3B in the supplemental material). Histological examinations indicated that the corium layer of OXA-treated N315-challenged BALB/c mice exhibited extensive inflammation with leukocyte infiltration, more flake-like abscess formation, and destroyed skin structure compared with the OXA-treated N315Δ*lpl*-, PBS-treated N315-, and N315Δ*lpl*-infected mice (see Fig. S3C in the supplemental material). In contrast, the skin of OXA-treated N315Δ*lpl-*challenged mice displayed similar leukocyte infiltration and sporadic abscess formation relative as those of PBS-treated N315Δ*lpl*-infected mice. These pathological phenomena might be caused by β-lactam-induced MRSA Lpls, which stimulated higher levels of IL-6 and TNF-α in mice (see Fig. S3D and E in the supplemental material), thereby promoting an exuberant, systemic inflammatory response.

To further determine whether MRSA Lpl-promoted pathogenicity of MRSA is associated with TLR2, we subcutaneously challenged C57BL/6 and C57BL/6 TLR2^−/−^ mice in both flanks with N315 and N315Δ*lpl*. Results showed that skin abscesses in wild-type C57BL/6 mice caused by OXA-treated N315 were larger than those caused by OXA-treated N315Δ*lpl*, PBS-treated N315, and N315Δ*lpl* between 4 and 10 days postinfection (see Fig. S4A in the supplemental material). This observation was further shown in the photographs of skin abscesses of C57BL/6 mice at 7 days postinfection (see Fig. S4B in the supplemental material). N315 and N315Δ*lpl* caused larger skin lesions in C57BL/6 TLR2^−/−^ mice than those in C57BL/6 mice (Fig. 5E and F; see Fig. S4B and C in the supplemental material), consistent with the previous results (32). However, the skin abscesses in C57BL/6 TLR2^−/−^ mice caused by OXA-treated N315 were negligibly different from those caused by OXA-treated N315Δ*lpl*, PBS-treated N315, and N315Δ*lpl* (Fig. 5E and F; see Fig. S4B and C). Overall, these data confirmed that β-lactam-induced Lpls aggravated host TLR2-dependent MRSA infections.

10.1128/mBio.00880-19.4 FIG S4 Abscess formation in C57BL/6 and C57BL/6 TLR2^−/−^ mice after infection. Mice were injected subcutaneously with 5 × 10^7^ CFU of N315 and N315Δ*lpl* bacterial cells and intraperitoneally injected with 1 μg of OXA per gram of weight twice a day for 14 days. PBS-treated N315- and N315Δ*lpl*-challenged mice served as controls. (A) Abscess areas were measured daily. Number of mice used, *n *=* *5. Error bars indicate SD. Statistical significances were calculated by multiple *t* test. *, *P < *0.05; **, *P < *0.01; ***, *P < *0.001. (B) The abscesses in C57BL/6 mice 7 days after infection. The abscesses in mice caused by OXA-treated N315 were larger than those caused by OXA-treated N315Δ*lpl*, PBS-treated N315, and PBS-treated N315Δ*lpl*. (C) Abscesses in C57BL/6 TLR2^−/−^ mice 7 days after infection. The abscesses in C57BL/6 TLR2^−/−^ mice caused by OXA-treated N315 were negligibly different from those caused by OXA-treated N315Δ*lpl*, PBS-treated N315, and PBS-treated N315Δ*lpl*. Download FIG S4, TIF file, 2.8 MB. Copyright © 2019 Shang et al. 2019 Shang et al. This content is distributed under the terms of the Creative Commons Attribution 4.0 International license.

## DISCUSSION

MRSA is distinct from MSSA in terms of the acquisition of a genetic element called staphylococcal cassette chromosome *mec*, in which *mecA* encodes an alternative penicillin-binding protein 2a (PBP2a) with a low afﬁnity for β-lactams (24). Thus, MRSA strains are resistant to nearly all β-lactam antibiotics (3). As antibiotics, β-lactams bind to PBPs and inhibit the transpeptidation and transglycosylation of the cell wall, resulting in a weakened cell wall and inducing cell lysis and death (33). This type of antibiotic, particularly cephalosporins and β-lactam–β-lactamase inhibitor combinations, has been empirically used for clinical treatments of infectious diseases (34). Subinhibitory concentrations of antistaphylococcal agents may occur due to either antibiotic-resistant microorganisms or pharmacokinetics of antibiotics (12, 34). For MRSA infections, which are not initially recognized, β-lactams not only are ineffective in treatment but also possibly contribute to poor outcomes by enhancing the pathogenicity of MRSA. Nonetheless, the underlying mechanisms remain obscure (6). In addition to antimicrobial activity, signal induction may be implemented by subinhibitory concentrations of β-lactams, which actively promote S. aureus biofilm formation (10), induce PBP2a to reduce peptidoglycan cross-linking in MRSA (3), and enhance virulence factors, such as alpha-toxins, PVL, SpA, and enterotoxins (9, 35–37).

In this study, we showed that a three-gene constituent *lpl* cluster in the MRSA genome was upregulated in response to β-lactam induction. This *lpl* cluster was widely distributed among the major prevalent MRSA clones (see Table S5 in the supplemental material). Lpls could be upregulated after treatment with nearly all β-lactam antibiotics (Fig. 1A). β-Lactams can induce PVL expression in S. aureus by interfering with PBP1 and triggering SarA and Rot global regulators (9). Our results showed that deletion of SarA (N315Δ*sarA*) failed to upregulate *lpl* expression under OXA treatment (Fig. 2F), whereas N315Δ*agrA* and USA300Δ*agrA* showed *lpl* expression comparable to their wild-type strains, indicating that β-lactam-induced Lpl expression in MRSA is probably SarA controlled via an *agr-*independent pathway. EMSA data revealed the direct regulation of SarA during Lpl expression (Fig. 2B). However, further investigations should be performed to clarify how β-lactams trigger SarA expression.

10.1128/mBio.00880-19.9 TABLE S5 Distribution of *lpl* cluster in major MRSA clones. Download Table S5, DOCX file, 0.02 MB. Copyright © 2019 Shang et al. 2019 Shang et al. This content is distributed under the terms of the Creative Commons Attribution 4.0 International license.

In contrast to β-lactam-induced SpA and PVL, which exhibit a controversial pathogenic role in S. aureus (9), some Lpps of S. aureus are crucial players in alerting the host immune system by recognizing TLR2/TLR1 or TLR2/TLR6 receptors (38, 39). Proinflammatory cytokines were not induced by purified lipidated SA2275-his proteins in BMDM TLR2^−/−^ cells and C57BL/6 TLR2^−/−^ mice (Fig. 3C and D and Fig. 4C and D), suggesting that TLR2 is required by Lpls in stimulating the immune system. Although N315Δ*lpl* infections induced less IL-6 and TNF-α production in mice than did the wild-type strain, OXA-treated N315Δ*lpl-*infected mice still produced higher levels of IL-6 and TNF-α cytokines than did untreated wild-type strain-challenged mice (Fig. S3D and E), suggesting that other mechanisms might be involved in immune system modulation by β-lactam-treated MRSA. For instance, β-lactam-promoted PBP2a induction can diminish peptidoglycan cross-linking, thereby enhancing phagocytic degradation and detection and promoting IL-1β production (3).

Our study also demonstrated that increasing MRSA pathogenicity was attributed to β-lactam-induced Lpls (Fig. 5A, B, and D; Fig. S3B and C). A possible explanation is that the higher levels of IL-6 and TNF-α in mice induced by β-lactam-induced Lpls promoted exuberant, systemic inflammatory responses, thereby facilitating the pathogenicity of MRSA. Schmaler et al. (32) found that TLR2^−/−^ or MyD88^−/−^ mice showed more weight loss and higher bacterial loads in kidneys and knees after infection with S. aureus than C57BL/6 mice. Our results also revealed that N315 and N315Δ*lpl* strains caused more pronounced skin lesions in TLR2^−/−^ mice than in C57BL/6 mice (Fig. 5E and F; Fig. S4B and C). This may be attributed to the low levels of proinflammatory cytokines induced in TLR2^−/−^ mice, facilitating MRSA colonization and infection.

In conclusion, this work focused on the function and regulation of an *lpl* cluster in response to the induction of subinhibitory concentrations of β-lactams. β-Lactam-induced MRSA *lpl* expression is SarA dependent, and upregulation of *lpl* after β-lactam treatment is directly controlled by the global regulator SarA. We demonstrated that the increased Lpls in MRSA significantly promote TLR2-dependent signaling pathway activation and result in inflammatory response by triggering IL-6 and TNF-α levels *in vitro* and *in vivo*, thereby possibly contributing to bacterial pathogenicity by inducing host immune responses and promoting bacterial colonization. Our data support the recommendation to clinicians regarding the prudent usage of β-lactams, which possibly aggravate the clinical outcomes of MRSA infections.

## MATERIALS AND METHODS

### Ethics statement.

BALB/c mice were purchased from the Laboratory Animal Center of Army Medical University. C57BL/6 TLR2^−/−^ mice were provided as a gift by Yuzhang Wu at the Department of Immunology, Army Medical University. All animal experiments were approved by the Institutional Animal Care and Use Committee of Army Medical University (protocol no. SYXK-PLA-20120031). All animal experimental procedures were performed in accordance with the Regulations for the Administration of Affairs Concerning Experimental Animals approved by the State Council of the People’s Republic of China. Cervical dislocation was used as the euthanasia method for all experimental mice.

### Bacterial strains, plasmids, and primers.

Bacterial strains and plasmids used in this study are listed in Table S4 in the supplemental material. All primers used are listed in Table S6.

10.1128/mBio.00880-19.10 TABLE S6 Primers used in this study. Download Table S6, DOCX file, 0.03 MB. Copyright © 2019 Shang et al. 2019 Shang et al. This content is distributed under the terms of the Creative Commons Attribution 4.0 International license.

### Antibiotic susceptibility tests.

Antibiotic susceptibility was determined using broth microdilution methods according to the protocols recommended by the Clinical and Laboratory Standards Institute (CLSI) (40). The antibiotic susceptibility results for all strains are listed in Table S2.

### Preparation of recombinant lipidated SA2275-his, unlipidated SA2275-his (-sp), and SarA-his proteins.

The lipidated SA2275-his proteins were isolated from the membrane fraction of MRSA N315Δ*lpl* carrying expression vector pXR-*sa2275*-*his* (Table S4) as previously described (21). The endotoxin contamination in the purified lipidated SA2275-his stock was determined by a *Tachypleus* amebocyte lysate test (Horseshoe Crab Reagent Manufactory Co. Ltd., China), and a concentration of less than 0.08 endotoxin units (EU) was approved to be used for the stimulation of cytokine production by macrophages and animals (41).

pET28a-*sa2275* and pET28a-*sarA* were transformed into Escherichia coli BL21(DE3) for the expression of SA2275-his (-sp) and SarA-his fusion proteins (42), which were purified by Ni-NTA affinity chromatography and identified by Western blotting.

### Preparation of polyclonal antibodies against recombinant proteins.

Female BALB/c mice (6 to 8 weeks) were immunized subcutaneously with SA2275-his (-sp) or SarA-his recombinant proteins to prepare polyclonal antibodies (42).

### Preparation of total bacterial proteins and culture supernatant proteins.

The overnight culture of S. aureus strain was diluted 1:100 in brain heart infusion (BHI) medium with or without the addition of β-lactam antibiotics and cultivated at 37°C to an OD_600_ of 2.0. Then, bacterial cells in 3 ml culture were harvested, washed twice with PBS, and resuspended in 1 ml of cold PBS supplemented with 1% (mass/vol) β-mercaptoethanol (Sigma, USA) and 1 mM phenylmethylsulfonyl fluoride (PMSF) (Beyotime, China) on ice. Cells were broken by addition of 0.1-mm-diameter zirconia-silica beads with shaking on the Minibeadbeater 16 instrument (Biospec, USA). Proteins in 1 ml of the culture supernatant were precipitated with 7.5% (vol/vol) trichloroacetic acid (TCA)-0.2% (vol/vol) deoxycholic acid solution (43). The protein concentration was determined using the Bradford protein assay kit (Beyotime, China).

### Protein identification.

LC-MS/MS was performed to identify proteins induced by β-lactam antibiotics as previously described (42). The antibiotic-induced protein band was excised and analyzed through LC-MS/MS by using an UltiMate3000 RSLCnano-liquid chromatography/Bruker Maxis 4G Q-TOF instrument. The resulting peptide mass fingerprints were compared against the open reading frames (ORFs) of N315 by using Mascot and Mascot Daemon software (Matrix Science).

### RT-PCR and RT-qPCR.

Total RNA of MRSA N315 was extracted as previously described (44). RT-PCR was used to determine whether *sa2275*, *sa2274*, and *sa2273* were cotranscribed. RT-qPCR was performed to detect the expression levels of *lpl* genes (*sa2275*, *sa2274*, and *sa2273*) using SsoAdvanced Universal SYBR Green Supermix (Bio-Rad, USA). The relative expression level of all tested genes was normalized to that of the 16S rRNA gene.

### EMSA.

The predicted *lpl* cluster promoter, an AT-rich motif fragment (56 bp), was synthesized using primer pairs (EMSA-*lpl*^P^ fwd/EMSA-*lpl*^P^ rev) as described previously (45). The corresponding mutated GC-rich motif fragment was also synthesized by primer pair EMSA-*lpl*^PM^ fwd/EMSA-*lpl*^PM^ rev and served as controls. Ten picomoles of DNA fragment was incubated with a variable amount of recombinant SarA-his (0 to 240 pM) in a 20-μl reaction mixture containing 10 mM HEPES (pH 7.6), 1 mM EDTA, 2 mM dithiothreitol, 50 mM KCl, 0.05% (vol/vol) Triton X-100, and 5% (vol/vol) glycerol. Binding reaction mixtures were equilibrated for 20 min at room temperature before electrophoresis. Reaction mixtures were separated on 6% (mass/vol) native polyacrylamide gel electrophoresis in 0.5× TBE (Tris-boric acid-EDTA) buffer at 90 V for 2 h at 4°C. Gels were stained by GelRed dye (Biotium, USA) and observed under UV light.

### Construction of gene deletion mutant and overexpression strains.

The *lpl* cluster markerless deletion mutant was constructed using homologous recombinant strategy described previously (44). Briefly, pYT3-Δ*lpl* and pBT2-Δ*lpl* plasmids were used to construct *lpl* cluster markerless deletion mutant. The deletion of *lpl* cluster was confirmed by PCR and DNA sequencing. Similar strategies were used to construct N315Δ*sarA*, N315Δ*agrA*, USA300Δ*agrA*, and USA300Δ*lpl* mutant strains.

The pLI-*lpl* plasmid was electroporated into N315Δ*lpl* and USA300Δ*lpl* to generate *lpl* overexpression strains N315Δ*lpl*/pLI-*lpl* and USA300Δ*lpl*/pLI-*lpl*, respectively. A similar strategy was used to construct N315Δ*sarA*/pLI-*sarA*. The empty pLI50 plasmid-transformed N315Δ*lpl*, N315Δ*sarA*, and USA300Δ*lpl* strains served as controls.

### Cytokine determination.

For stimulation experiment, RAW 264.7 cells (10^6^/well) were infected with MRSA strains (multiplicity of infection [MOI] of 30) in a 24-well microtiter plate for 6 h as previously described (46). Then, the supernatant was collected, and the levels of IL-6 and TNF-α were determined with an ELISA kit according to the manufacturer’s instructions (R&D Systems, USA).

BMDMs were isolated from 12-week-old female wild-type C57BL/6 or C57BL/6 TLR2^−/−^ mice as described previously (47). Briefly, bone marrow cells were obtained from femurs by flushing with complete RPMI 1640 medium (HyClone). After removed of red blood cells, the cells were cultured on 6-well plates with RPMI 1640 containing 10% (vol/vol) heat-inactivated fetal bovine serum (FBS), 20 ng/ml macrophage colony-stimulating factor (M-CSF), 100 U/ml penicillin, and 100 μg/ml streptomycin at 37°C to a fluent monolayer. Cells were identified by the BD FACS Canto-II flow cytometer (BD Biosciences, USA) (48). BMDMs were seeded at a density of 5 × 10^5^ cells/well in 24-well plates and allowed to adhere overnight followed by stimulation with lipidated SA2275-his proteins for 6 h, and the levels of IL-6 and TNF-α were measured by ELISA.

Female BALB/c mice were infected via tail vein injection with 1 × 10^7^ CFU of the MRSA strain of interest for 6 h. To detect the cytokine induction capacity of purified lipidated SA2275-his proteins *in vivo*, female C57BL/6 and C57BL/6 TLR2^−/−^ mice were challenged via tail vein injection with 50, 100, and 500 ng recombinant SA2275-his proteins for 6 h, respectively. Blood samples were collected 6 h postinjection, and the levels of IL-6 and TNF-α in mouse sera were determined by ELISA.

### Animal experiments.

BALB/c mice were randomly divided into two groups and infected via tail vein injection with 1 × 10^7^ CFU of the GFP expression plasmid (pGFP)-transformed N315 or N315Δ*lpl* and sacrificed 5 days after infection. Mouse organs (i.e., heart, lung, liver, spleen, and kidney) were isolated and subjected to the determination of GFP fluorescence efficiency in organs with the IVIS Lumina LT system and analyzed by Living Image 4.4 Software. The bacterial loads in the infected kidneys were also counted via plate dilution assay as described previously (43).

For skin abscess formation, BALB/c, C57BL/6, and C57BL/6 TLR2^−/−^ mice were fully anesthetized with 1% (mass/vol) pentobarbital sodium (50 mg/kg of body weight), and the back hair was depilated completely with 6% (mass/vol) sodium sulfide. Then, mice were subcutaneously inoculated with 5 × 10^7^ CFU of MRSA N315 and N315Δ*lpl* in both flanks of the murine back as described previously (49) and then randomly divided into two groups. The mice of the treatment group were intraperitoneally injected with 1 μg of OXA per gram of weight twice a day for 14 days. The PBS-injected mice served as controls. The abscess area assessed by the maximal length by width of the developing ulcer was measured daily.

### Statistical analysis.

Statistical analysis was carried out using GraphPad Prism 6.0. Replicate numbers and statistical tests for each experiment are listed in the figure legends.
