# Supplementary material for: β-Lactam Antibiotics Enhance the Pathogenicity of Methicillin-Resistant Staphylococcus aureus via SarA-Controlled Lipoprotein-Like Cluster Expression
Source: mBio. 2019 Jun 11;10(3):e00880-19. doi: 10.1128/mBio.00880-19 (PMC6561022; doi:10.1128/mBio.00880-19)
Supplement: TABLE S1 [file mBio.00880-19-st001.docx]

**TABLE S1** Predicted Lpps of *S. aureus* N315.

| **No** | **Locus tag** | **Function/Annotation** | **PFAM** | **SP** ^a^ | **Lipobox** | **Mass**  **(kDa)** | **Dissemination** | **References** |  |
| --- | --- | --- | --- | --- | --- | --- | --- | --- | --- |
|  |  | **Fe transport** |  |  |  |  |  |  |  |
| 1 | SA2079 *fhuD2* | Fe ABC transporter/FhuD2 | Peripla_BP_2, ABC2_membrane_3 | 17 | LAA C | 34 | 16 | ([1](#_ENREF_1), [2](#_ENREF_2)); |  |
| 2 | SA0691 | Transferrin receptor/SstD | Peripla_BP_2 | 18 | LAA C | 38 | 14 | ([3](#_ENREF_3)) |  |
| 3 | SA0980 isdE | Fe ABC transporter/IsdE | Peripla_BP_2 | 19 | LTS C | 33 | 10 | ([4-6](#_ENREF_4)); |  |
| 4 | SA0331 | FepA, Fe binding protein, part of fepABC and tat-AC cluster | Peptidase_M75 | 17 | IAA **C** | 32 | 10 | ([7](#_ENREF_7)) |  |
| 5 | SA1979 | Fe ABC transporter | Peripla_BP_2 | 21 | VAA C | 37 | 10 |  |  |
| 6 | SA0891 | Fe/B12 ABC_transptr_periplasmic_BD | Peripla_BP_2 | 21 | VAG C | 36 | 14 |  |  |
| 7 | SA0217 | Iron Binding Protein | SBP_bac_1, 6, 8, 11 | 17 | LSS C | 37 | 4* |  |  |
| 8 | SA0566 | Fe ABC transporter | Peripla_BP_2 | 18 | LSG C | 33 | 5* |  |  |
|  |  | **Other cation transport** |  |  |  |  |  |  |  |
| 9 | SA0587 | Manganese-binding protein MntC (SitC) | ZnuA, Nit_Regul_Hom | 17 | VAA C | 35 | 19 | ([8-10](#_ENREF_8)) |  |
| 10 | SA2194 | Zinc-binding, adcA-like | ZnuA, ZinT | 20 | LAA C | 59 | 10 |  |  |
|  |  |  |  |  |  |  |  | *(Continued)* |  |
| **No** | **Locus tag** | **Function/Annotation** | **PFAM** | **SP** ^a^ | **Lipobox** | **Mass**  **(kDa)** | **Dissemination** | **References** |  |
|  |  | **Other cation transport** |  |  |  |  |  |  |  |
| 11 | SA2255 *opp-1A* | Cobalt and nickel transporter Cnt (Opp1A) | SBP_bac_5 | 20 | LTG C | 60 | 10 | ([11](#_ENREF_11)) |  |
| 12 | SA0229 | Nickel ABC transporter | SBP_bac_5 | 18 | LSG C | 55 | 10^+^ |  |  |
| 13 | SA2074 *modA* | Molybdenum ABC transporter (ModA) | SBP_bac_11, 1, PBP_like_2 | 19 | LAG C | 29 | 15 | ([12](#_ENREF_12)) |  |
|  |  | **Anion transport** |  |  |  |  |  |  |  |
| 14 | SA1221 | Phosphate ABC transporter | PBP_like_2, | 20 | LGA C | 36 | 15 |  |  |
|  |  | **AA and Peptide transport** |  |  |  |  |  |  |  |
| 15 | SA2235 *opuCC* | Glycine betaine /carnitine/ choline ABC transporter (OpuCc) | OpuAC | 20 | LSG C | 35 | 19 |  |  |
| 16 | SA2202 | Amino acid ABC transporter | SBP_bac_3 | 17 | LAA C | 29 | 12 |  |  |
| 17 | SA0849 | Oligopeptide ABC transporter (Opp3A) | SBP_bac_5 | 20 | LSG C | 62 | 11 | ([13](#_ENREF_13)) |  |
| 18 | SA0850 | Oligopeptide ABC transporter (Opp4A) | SBP_bac_5 | 20 | LSA C | 65 | 5 | ([13](#_ENREF_13)) |  |
| 19 | SA0422 | NLPA/ D Methionine binding (GmpC) | Lipoprotein_9 (NLPA) | 17 | LAA **C** | 30 | 9 | ([14](#_ENREF_14)) |  |
|  |  |  |  |  |  |  |  | *(Continued)* |  |
| **No** | **Locus tag** | **Function/Annotation** | **PFAM** | **SP** ^a^ | **Lipobox** | **Mass**  **(kDa)** | **Dissemination** | **References** |  |
|  |  | **AA and Peptide transport** |  |  |  |  |  |  |  |
| 20 | SA0771 | D-Methionine ABC transporter | OpuAC, Lipoprotein_9 | 19 | LAA C | 30 | 15 |  |  |
|  |  |  |  |  |  |  |  |  |  |
|  |  | **Biosynthesis** |  |  |  |  |  |  |  |
| 21 | SA1719 | CamS sex pheromone biosynthesis | CamS | 17 | LAA **C** | 45 | 14 |  |  |
|  |  | **Respiration** |  |  |  |  |  |  |  |
| 22 | SA0913 | Quinol oxidase, subunit II (QoxA) | COX2 | 19 | LSG C | 41 | 21 |  |  |
| 23 | SA0663 | Electron transfer domain/SaeP | CfAFP, DM13 | 20 | LGA C | 16 | 22 | ([15](#_ENREF_15)) |  |
|  |  | **Chaperone-Foldases** |  |  |  |  |  |  |  |
| 24 | SA1659 *prsA* | Foldase protein PrsA | Rotamase | 20 | LGA C | 36 | 15 | ([16](#_ENREF_16), [17](#_ENREF_17)) |  |
| 25 | SA2197 | Thioredoxin/Protein disulfide-isomerase | Thioredoxin_2, 4, 5 |  | LTA C | 23 | 15* |  |  |
|  |  | **Protein translocation** |  |  |  |  |  |  |  |
| 26 | SA1893 | YidC (OxaA)–essential protein | OATP, 60KD_IMP | 19 | LAG C | 34 | 25 |  |  |
|  |  | **Lpl cluster** |  |  |  |  |  |  |  |
| 27 | SA0396 *lpl1* | Lpl-1 νSaα specific | DUF576 | 32 | IAG C | 31 | + | ([18](#_ENREF_18)) |  |
| 28 | SA0397 *lpl2* | Lpl-2 νSaα specific | DUF576 | 22 | IIG C | 31 | + | ([18](#_ENREF_18)) |  |
| 29 | SA0398 *lpl3* | Lpl-3 νSaα specific | DUF576 | 23 | IAG C | 30 | + | ([18](#_ENREF_18)) |  |
| 30 | SA0400 *lpl4* | Lpl-4 νSaα specific | DUF576 | 22 | IIG C | 30 | + | ([18](#_ENREF_18)) |  |
| 31 | SA0401 *lpl5* | Lpl-5 νSaα specific | DUF576 | 22 | VAG C | 30 | + | ([18](#_ENREF_18)) |  |
|  |  |  |  |  |  |  |  | *(Continued)* |  |
| **No** | **Locus tag** | **Function/Annotation** | **PFAM** | **SP** ^a^ | **Lipobox** | **Mass**  **(kDa)** | **Dissemination** | **References** |  |
|  |  | **Lpl cluster** |  |  |  |  |  |  |  |
| 32 | SA0402 *lpl6* | Lpl-6 νSaα specific | DUF576 | 23 | IIG C | 31 | + | ([18](#_ENREF_18)) |  |
| 33 | SA0403 *lpl7* | Lpl-7 νSaα specific | DUF576 | 22 | IIG C | 31 | + | ([18](#_ENREF_18)) |  |
| 34 | SA0404 *lpl8* | Lpl-8 νSaα specific | DUF576 | 22 | ATS C | 30 | + | ([18](#_ENREF_18)) |  |
| 35 | SA0405 *lpl9* | Lpl-9 νSaα specific | DUF576 | 23 | IGG C | 32 | + | ([18](#_ENREF_18)) |  |
| 36 | SA2273 | Tandem lpp | DUF576 | 23 | IGG C | 31 | 3* |  |  |
| 37 | SA2274 | Tandem lpp | DUF576 |  |  | 30 | + |  |  |
| 38 | SA2275 | Tandem lpp | DUF576 | 23 | IGA C | 30 | + |  |  |
|  |  | **Unknown function** |  |  |  |  |  |  |  |
| 39 | SA0359 | Unknown function | PepSY | 18 | LTA C | 21 | 17 |  |  |
| 40 | SA0363 | Unknown function | DUF1748 | 19 | LTG C | 24 | 17* |  |  |
| 41 | SA1361 | Unknown function |  | 16 | LAG **C** | 21 | 14 |  |  |
| 42 | SA0943 | Cell-wall binding lipoprotein | YkyA, EzrA | 19 | LAG C | 24 | 15* |  |  |
| 43 | SA2247 | Unknown function | DUF1307 | 20 | LSA C | 17 | 13 |  |  |
| 44 | SA0695 | Unknown function | IncA, TarH | 18 | ISA C | 34 | 12* |  |  |
| 45 | SA2158 | Unknown function | PA26, IncA, CLN3,  DUF1510 | 17 | LAA C | 23 | 12 |  |  |
| 46 | SA2473 | Unknown function | DUF_1980 | 20 | LYS C | 44 | 11* |  |  |
| 47 | SA0632 | Unknown function | PA26, IncA | 17 | LTG C | 15 | 6 |  |  |
| 48 | SA1056 | Unknown function | FAM176 | 18 | VAG C | 36 | 5 |  |  |
| 49 | SA0291 | Unknown function | DUF4467 | 17 | LAG C | 15 | 5* |  |  |
| 50 | SA0739 | Unknown function | DUF5067 | 17 | LGA C | 28 | 5* |  |  |
| 51 | SA2198 | Uncharacterized protein | DUF4467 | 17 | ISG **C** | 14 | + |  |  |
|  |  |  |  |  |  |  |  | *(Continued)* |  |
| **No** | **Locus tag** | **Function/Annotation** | **PFAM** | **SP** ^a^ | **Lipobox** | **Mass**  **(kDa)** | **Dissemination** | **References** |  |
|  |  | **Unknown function** |  |  |  |  |  |  |  |
| 52 | SA1317 | Unknown function | DUF1672 | 17 | LSG C | 36 | 6 |  |  |
| 53 | SA1318 | Unknown function | DUF1672 | 17 | LSG C | 34 | 6 |  |  |
| 54 | SA1319 | Unknown function | DUF1672 | 17 | LSG C | 34 | 6 |  |  |
| 55 | SA1640 | Unknown function | PA26 LPAM_1 | 17 | LVA C | 19 | 7* |  |  |
| 56 | SA1619 | Unknown function | [DUF1510](http://www.genome.jp/dbget-bin/www_bget?pfam:DUF1510) | 18 | LTA C | 24 | 5* |  |  |
| 57 | SA1616 | Unknown function | DUF4909 | 24 | LSS C | 19 | 5* |  |  |

^a^ The number of staphylococcal species in which the corresponding homologous gene/protein (more than 40% identity) was present.

+ *S. aureus* specific gene.

*Staphylococcal specific gene.

**REFERENCES**

1. Mariotti P, Malito E, Biancucci M, Lo Surdo P, Mishra RP, Nardi-Dei V, Savino S, Nissum M, Spraggon G, Grandi G, Bagnoli F, Bottomley MJ. 2013. Structural and functional characterization of the *Staphylococcus aureus* virulence factor and vaccine candidate FhuD2. Biochem J 449:683-93.

2. Sebulsky MT, Heinrichs DE. 2001. Identification and characterization of fhuD1 and fhuD2, two genes involved in iron-hydroxamate uptake in *Staphylococcus aureus*. J Bacteriol 183:4994-5000.

3. Morrissey JA, Cockayne A, Hill PJ, Williams P. 2000. Molecular cloning and analysis of a putative siderophore ABC transporter from *Staphylococcus aureus*. Infect Immun 68:6281-8.

4. Grigg JC, Vermeiren CL, Heinrichs DE, Murphy ME. 2007. Heme coordination by *Staphylococcus aureus* IsdE. J Biol Chem 282:28815-22.

5. Mazmanian SK, Ton-That H, Su K, Schneewind O. 2002. An iron-regulated sortase anchors a class of surface protein during *Staphylococcus aureus* pathogenesis. Proc Natl Acad Sci U S A 99:2293-8.

6. Mazmanian SK, Skaar EP, Gaspar AH, Humayun M, Gornicki P, Jelenska J, Joachmiak A, Missiakas DM, Schneewind O. 2003. Passage of heme-iron across the envelope of *Staphylococcus aureus*. Science 299:906-9.

7. Biswas L, Biswas R, Nerz C, Ohlsen K, Schlag M, Schafer T, Lamkemeyer T, Ziebandt AK, Hantke K, Rosenstein R, Gotz F. 2009. Role of the twin-arginine translocation pathway in Staphylococcus. J Bacteriol 191:5921-9.

8. Cockayne A, Hill PJ, Powell NB, Bishop K, Sims C, Williams P. 1998. Molecular cloning of a 32-kilodalton lipoprotein component of a novel iron-regulated *Staphylococcus epidermidis* ABC transporter. Infect Immun 66:3767-74.

9. Diep BA, Phung Q, Date S, Arnott D, Bakalarski C, Xu M, Nakamura G, Swem DL, Alexander MK, Le HN, Mai TT, Tan MW, Brown EJ, Nishiyama M. 2014. Identifying potential therapeutic targets of methicillin-resistant *Staphylococcus aureus* through in vivo proteomic analysis. J Infect Dis 209:1533-41.

10. Muller P, Muller-Anstett M, Wagener J, Gao Q, Kaesler S, Schaller M, Biedermann T, Gotz F. 2010. The *Staphylococcus aureus* lipoprotein SitC colocalizes with Toll-like receptor 2 (TLR2) in murine keratinocytes and elicits intracellular TLR2 accumulation. Infect Immun 78:4243-50.

11. Remy L, Carriere M, Derre-Bobillot A, Martini C, Sanguinetti M, Borezee-Durant E. 2013. The *Staphylococcus aureus* Opp1 ABC transporter imports nickel and cobalt in zinc-depleted conditions and contributes to virulence. Mol Microbiol 87:730-43.

12. Neubauer H, Pantel I, Lindgren PE, Gotz F. 1999. Characterization of the molybdate transport system ModABC of *Staphylococcus carnosus*. Arch Microbiol 172:109-15.

13. Hiron A, Borezee-Durant E, Piard JC, Juillard V. 2007. Only one of four oligopeptide transport systems mediates nitrogen nutrition in *Staphylococcus aureus*. J Bacteriol 189:5119-29.

14. Williams WA, Zhang RG, Zhou M, Joachimiak G, Gornicki P, Missiakas D, Joachimiak A. 2004. The membrane-associated lipoprotein-9 GmpC from *Staphylococcus aureus* binds the dipeptide GlyMet via side chain interactions. Biochemistry 43:16193-202.

15. Makgotlho PE, Marincola G, Schafer D, Liu Q, Bae T, Geiger T, Wasserman E, Wolz C, Ziebuhr W, Sinha B. 2013. SDS interferes with SaeS signaling of *Staphylococcus aureus* independently of SaePQ. PLoS One 8:e71644.

16. Heikkinen O, Seppala R, Tossavainen H, Heikkinen S, Koskela H, Permi P, Kilpelainen I. 2009. Solution structure of the parvulin-type PPIase domain of *Staphylococcus aureus* PrsA--implications for the catalytic mechanism of parvulins. BMC Struct Biol 9:17.

17. Jousselin A, Renzoni A, Andrey DO, Monod A, Lew DP, Kelley WL. 2012. The posttranslocational chaperone lipoprotein PrsA is involved in both glycopeptide and oxacillin resistance in *Staphylococcus aureus*. Antimicrob Agents Chemother 56:3629-40.

18. Nguyen MT, Kraft B, Yu W, Demircioglu DD, Hertlein T, Burian M, Schmaler M, Boller K, Bekeredjian-Ding I, Ohlsen K, Schittek B, Gotz F. 2015. The νSaα specific lipoprotein like cluster (*lpl*) of *S. aureus* USA300 contributes to immune stimulation and invasion in human cells. PLoS Pathog 11:e1004984.
