# Supplementary figures and images for: β-Lactam Antibiotics Enhance the Pathogenicity of Methicillin-Resistant Staphylococcus aureus via SarA-Controlled Lipoprotein-Like Cluster Expression
Source: mBio. 2019 Jun 11;10(3):e00880-19. doi: 10.1128/mBio.00880-19 (PMC6561022; doi:10.1128/mBio.00880-19)

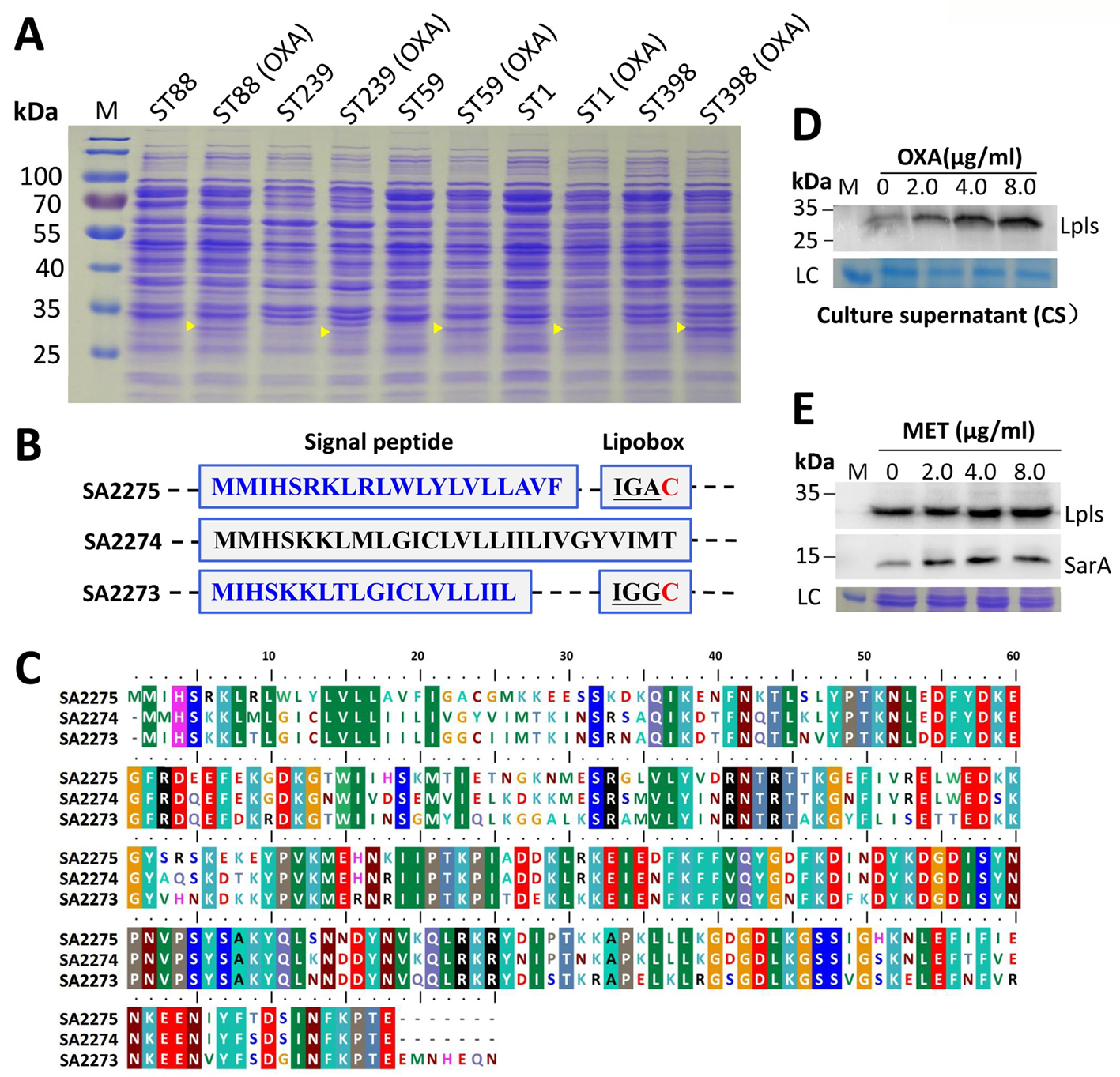

Supplement: FIG S1 [file mBio.00880-19-sf001.tif]

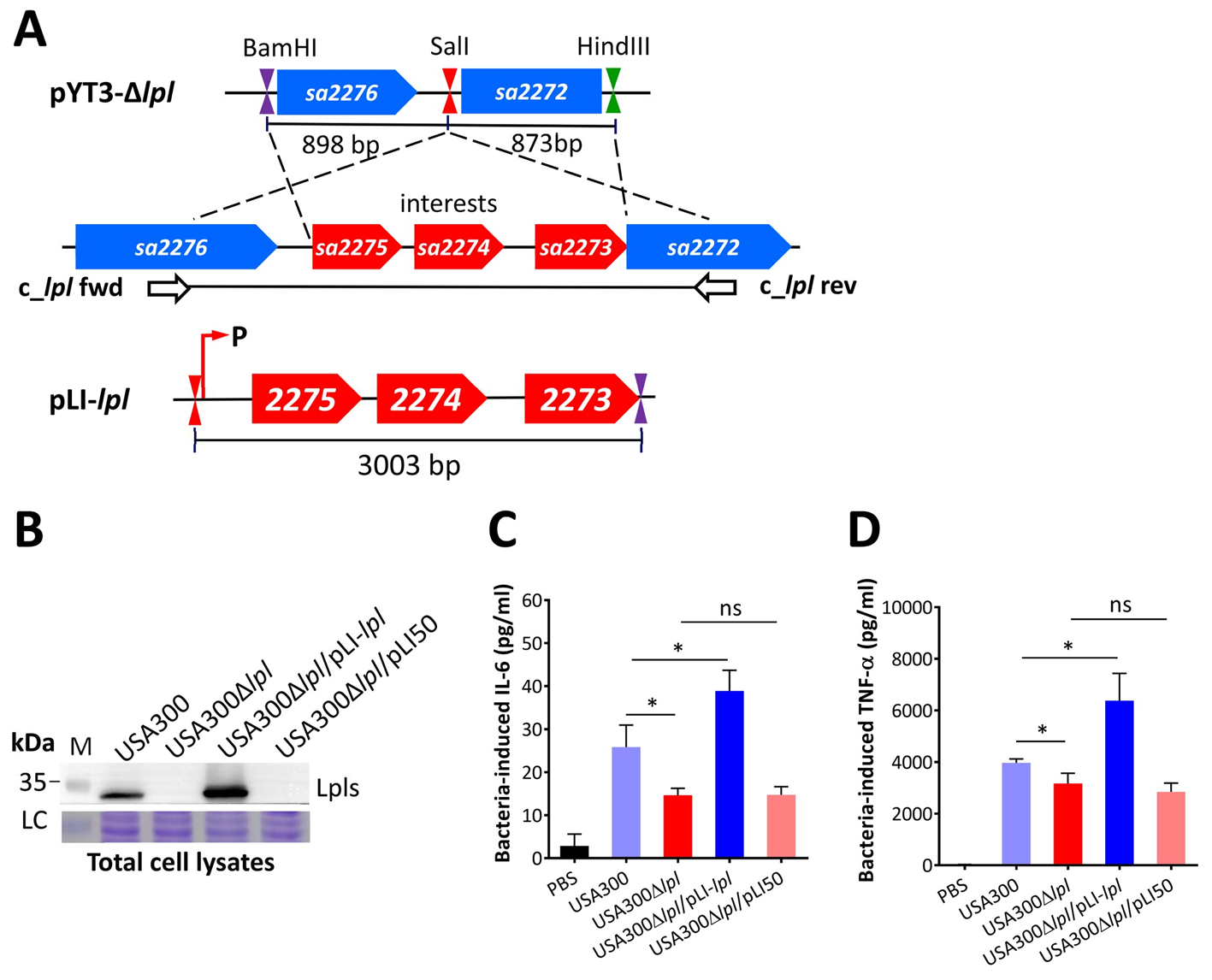

Supplement: FIG S2 [file mBio.00880-19-sf002.tif]

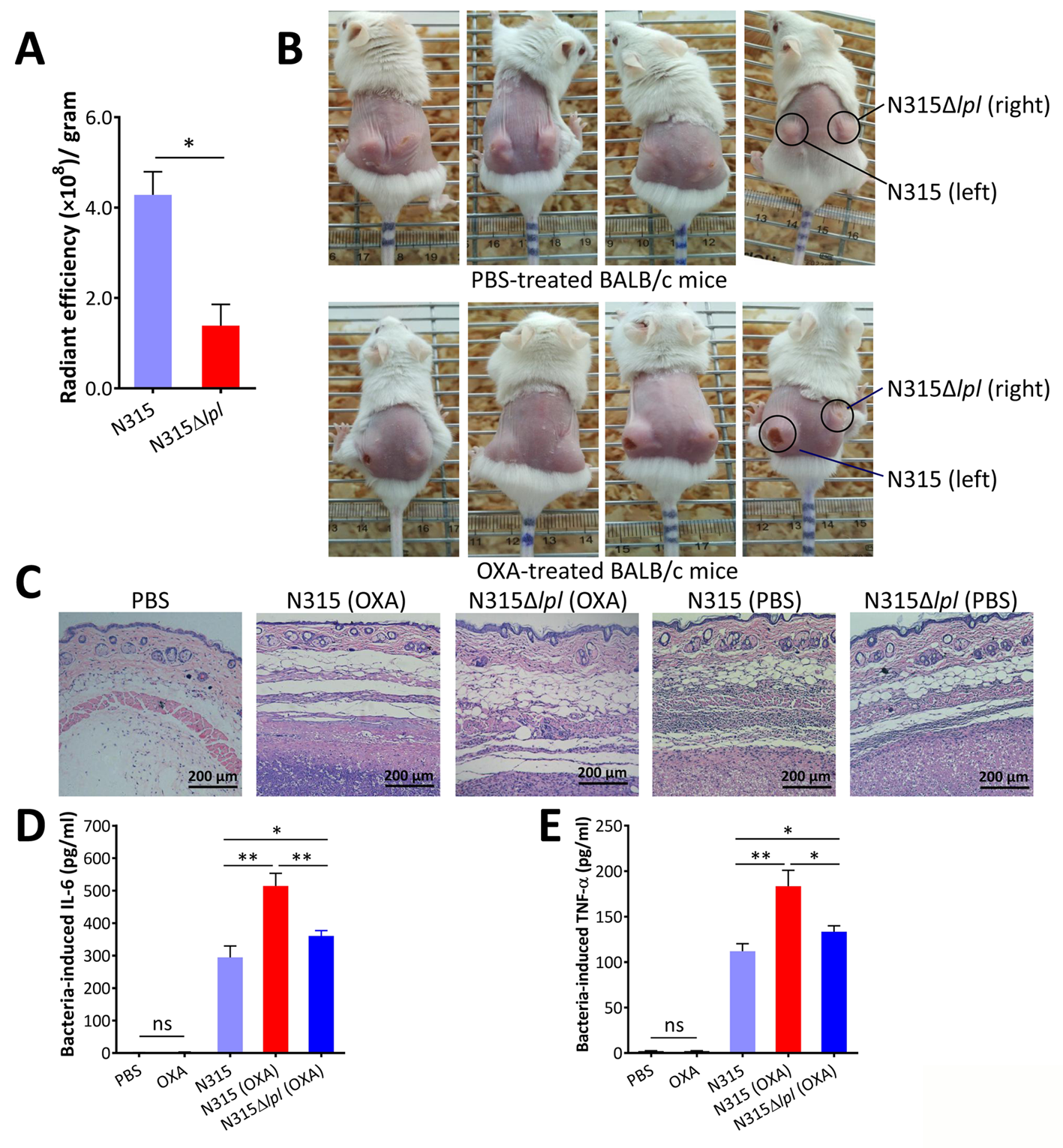

Supplement: FIG S3 [file mBio.00880-19-sf003.tif]

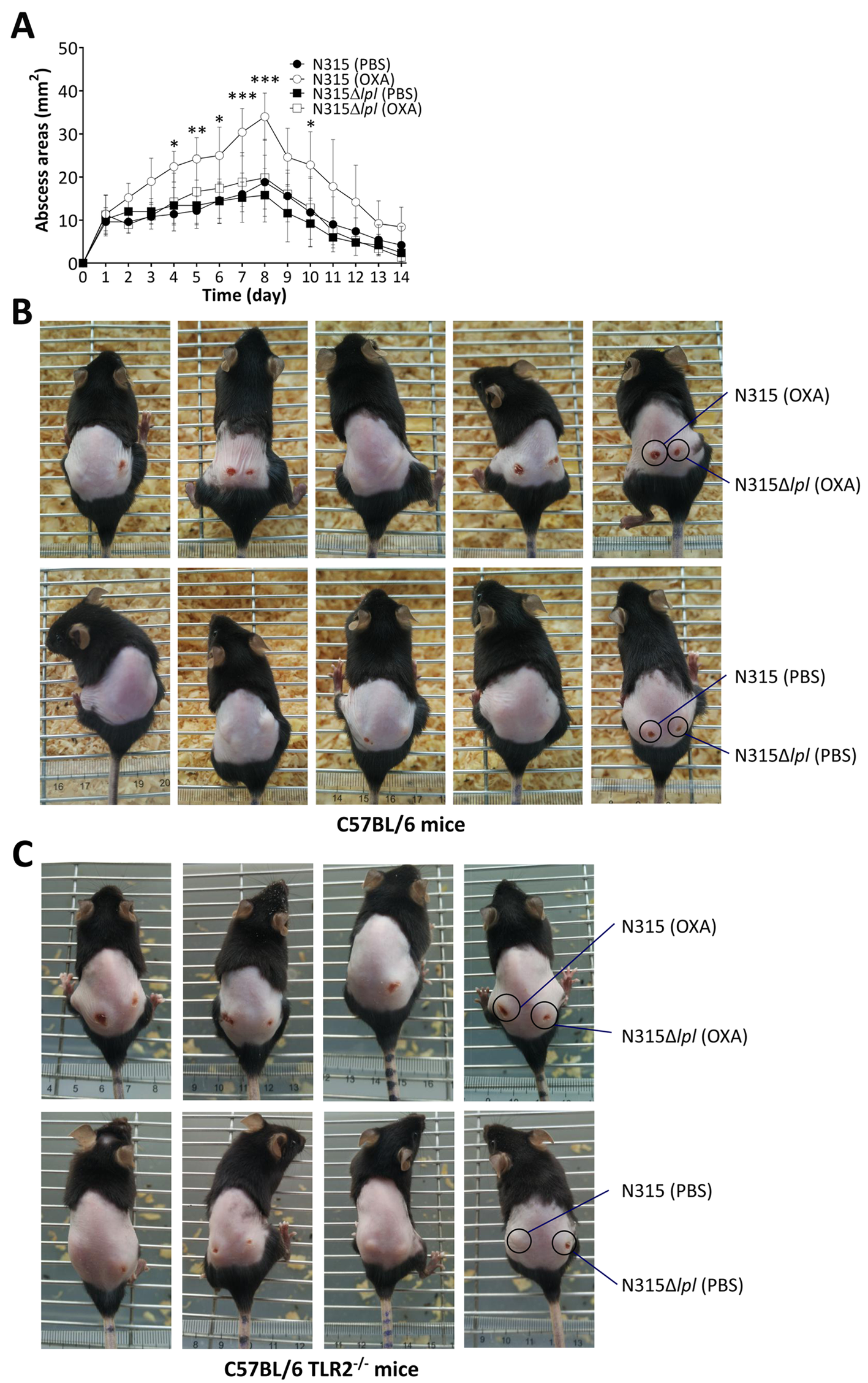

Supplement: FIG S4 [file mBio.00880-19-sf004.tif]
