# Supplementary material for: β-Lactam Antibiotics Enhance the Pathogenicity of Methicillin-Resistant Staphylococcus aureus via SarA-Controlled Lipoprotein-Like Cluster Expression
Source: mBio. 2019 Jun 11;10(3):e00880-19. doi: 10.1128/mBio.00880-19 (PMC6561022; doi:10.1128/mBio.00880-19)
Supplement: TABLE S2 [file mBio.00880-19-st002.docx]

**TABLE S2** The MICs of MRSA strains.

| **Strains** | **MIC (the subinhibitory concentrations of antibiotics used) (μg/ml) *^a^*** | | | | | | | | |
| --- | --- | --- | --- | --- | --- | --- | --- | --- | --- |
|  | OXA*^b^* | MET | FOX | IMI | MER | CHL | VAN | KAN | ERY |
| ST5 (N315) | 512 (2) | 8 (2) | 8 (2) | 4 (1) | 4 (1) | 8 (2) | 1 (0.25) | 256 (64) | 256(64) |
| ST88 (CQ200) | 32 (2) |  |  |  |  |  |  |  |  |
| ST239 (XN108) | 128 (2) |  |  |  |  |  |  |  |  |
| ST59 (CQ19) | 32 (2) |  |  |  |  |  |  |  |  |
| ST1 (SH256) | 32 (2) |  |  |  |  |  |  |  |  |
| ST398 (Ur300) | 8 (2) |  |  |  |  |  |  |  |  |

*^a^* The determination of MIC for each strain was repeated in thrice. OXA, oxacillin; MET, methicillin; FOX, cefoxitin; IMI, imipenem; MER, meropenem; CHL, chloramphenicol; VAN, vancomycin; KAN, kanamycin; ERY, erythromycin.

*^b^* The 1/4 MIC was used as the sub-inhibitory concentration of antibiotic in our study. However, the growth of MRSA N315 was significantly inhibited by OXA of more than 4 μg/ml, thereby, the sub-inhibitory concentration of OXA, 2 μg/ml, was used unless specifically stated.
