# Supplementary material for: β-Lactam Antibiotics Enhance the Pathogenicity of Methicillin-Resistant Staphylococcus aureus via SarA-Controlled Lipoprotein-Like Cluster Expression
Source: mBio. 2019 Jun 11;10(3):e00880-19. doi: 10.1128/mBio.00880-19 (PMC6561022; doi:10.1128/mBio.00880-19)
Supplement: TABLE S3 [file mBio.00880-19-st003.docx]

**TABLE S3** Proteins identified in the protein band of β-lactams induced MRSA N315 by LC-MS/MS.

| **NO.** | **Accession No** | **Protein name** | **Gene name** | **Score** | **Coverage** | **MW [kDa]** | **calc. pI** |
| --- | --- | --- | --- | --- | --- | --- | --- |
| 1 | BAB43578.1 | hypothetical protein | SA2275 | 188.81 | 55.43 | 30.4 | 8.9 |
| 2 | BAB42143.1 | naphthoate synthase | menB | 187.78 | 91.94 | 30.4 | 5.6 |
| 3 | BAB43576.1 | hypothetical protein | SA2273 | 173.39 | 89.77 | 30.8 | 9.3 |
| 4 | BAB42839.1 | D-alanine aminotransferase | SA1571 | 165.23 | 90.43 | 31.9 | 5.0 |
| 5 | BAB42325.1 | malonyl CoA-acyl carrier protein transacylase | FabD | 147.62 | 90.26 | 33.6 | 5.0 |
| 6 | BAB42593.1 | conserved hypothetical protein | SA1331 | 146.99 | 88.41 | 33.4 | 5.4 |
| 7 | BAB42190.1 | pyruvate dehydrogenase E1 component beta subunit | pdhB | 138.34 | 75.69 | 35.2 | 4.7 |
| 8 | BAB41256.1 | penicillin binding protein 2 prime | mecA | 136.20 | 68.26 | 76.1 | 8.7 |
| 9 | BAB41851.1 | conserved hypothetical protein | SA0618 | 134.05 | 68.78 | 23.7 | 4.7 |
| 10 | BAB42874.1 | plant metabolite dehydrogenase homolog | SA1606 | 132.72 | 90.61 | 31.4 | 5.1 |
| 11 | BAB42609.1 | branched-chain alpha-keto acid dehydrogenase E1 | bfmBAB | 130.79 | 70.34 | 36.0 | 4.8 |
| 12 | BAB42564.1 | menaquinone biosynthesis methyltransferase | gerCB | 128.43 | 84.23 | 27.4 | 8.7 |
| 13 | BAB41701.1 | cysteine synthase (o-acetylserine sulfhydrylase) homologue | cysK | 127.41 | 96.13 | 33.0 | 5.5 |
| 14 | BAB43704.1 | fructose-bisphosphate aldolase homolog | SA2399 | 127.29 | 90.88 | 33.0 | 5.0 |
| 15 | BAB41234.1 | DNA-binding response regulator | vicR | 125.51 | 73.19 | 27.4 | 5.2 |
| 16 | BAB41769.1 | hypothetical protein, similar to phosphomethylpyrimidine kinase, thiD homologue | SA0537 | 125.48 | 69.93 | 29.8 | 5.0 |
| 17 | BAB42295.1 | aspartate transcarbamoylase chain A | pyrB | 124.04 | 61.43 | 33.2 | 5.9 |
| 18 | BAB41727.1 | 50S ribosomal protein L1 | rplA | 122.23 | 74.35 | 24.7 | 9.0 |
| 19 | BAB42852.1 | lysophospholipase homolog | SA1584 | 120.03 | 85.82 | 31.8 | 5.7 |
| 20 | BAB42418.1 | hypothetical protein | SA1161 | 118.37 | 73.94 | 32.8 | 7.0 |
| 21 | BAB41440.1 | formate acetyltransferase | pflB | 115.86 | 87.18 | 84.8 | 5.5 |
| 22 | BAB43245.1 | conserved hypothetical protein | SA1957 | 111.37 | 94.39 | 32.3 | 5.4 |
| 23 | BAB42691.1 | 5'-methylthioadenosine nucleosidase/S-adenosylhomocysteine nucleosidase | pfs | 106.32 | 83.77 | 24.5 | 4.9 |
| 24 | BAB41748.1 | conserved hypothetical protein | SA0517 | 105.64 | 81.31 | 31.8 | 5.0 |
| 25 | BAB41937.1 | conserved hypothetical protein | SA0704 | 105.26 | 73.96 | 32.0 | 5.0 |
| 26 | BAB42110.1 | trans-2-enoyl-ACP reductase | fabI | 101.90 | 93.75 | 28.0 | 5.9 |
|  |  |  |  |  |  | *(Continued)* | |
| **NO.** | **Accession No** | **Protein name** | **Gene name** | **Score** | **Coverage** | **MW [kDa]** | **calc. pI** |
| 27 | BAB43371.1 | probable molybdate-binding protein | modA | 101.71 | 71.15 | 29.1 | 9.2 |
| 28 | BAB42350.1 | transcription pleiotropic repressor codY | codY | 101.17 | 79.38 | 28.7 | 6.2 |
| 29 | BAB41689.1 | 50S ribosomal protein L25 | rplY | 100.04 | 74.19 | 23.8 | 4.4 |
| 30 | BAB43588.1 | hypothetical protein, similar to accumulation-associated protein | SA2285 | 99.81 | 84.01 | 48.9 | 8.6 |
| 31 | BAB41594.1 | hypothetical protein, similar to nitro/flavin reductase | SA0367 | 99.23 | 69.32 | 28.6 | 5.2 |
| 32 | BAB42974.1 | methionyl aminopeptidase map | map | 99.14 | 85.32 | 27.5 | 5.3 |
| 33 | BAB42625.1 | conserved hypothetical protein | SA1363 | 98.10 | 61.23 | 31.9 | 5.5 |
| 34 | BAB43577.1 | hypothetical protein | SA2274 | 97.87 | 81.08 | 30.1 | 9.0 |
| 35 | BAB41972.1 | conserved hypothetical protein | SA0739 | 89.71 | 86.78 | 28.4 | 6.8 |
| 36 | BAB43615.1 | D-specific D-2-hydroxyacid dehydrogenase | ddh | 84.25 | 60.4 | 39.3 | 5.6 |
| 37 | BAB41519.1 | 5'-nucleotidase, lipoprotein e(P4) family | SA0295 | 75.70 | 64.53 | 33.3 | 9.5 |
| 38 | BAB41652.1 | PBP2_lipoprotein_GmpC | SA0422 | 70.34 | 71.43 | 30.5 | 9.0 |
| 39 | BAB41819.1 | lipoprotein, Streptococcal adhesin PsaA homologue | SA0587 | 68.21 | 77.99 | 34.7 | 8.7 |
| 40 | BAB41707.1 | conserved hypothetical protein | SA0477 | 62.39 | 69.37 | 32.0 | 5.2 |
| 41 | BAB41441.1 | formate acetyltransferase activating enzyme | pflA | 46.02 | 86.06 | 28.5 | 6.0 |
| 42 | BAB42501.1 | MoxR-like ATPase [General function prediction | SA1241 | 32.39 | 66.54 | 29.4 | 5.1 |
| 43 | BAB42288.1 | Ile-tRNA synthetase | ileS | 31.90 | 57.69 | 104.8 | 5.5 |
| 44 | BAB41267.1 | O-nucleotidylltransferase(9) | ant(9) | 29.51 | 57.69 | 29.0 | 8.0 |
| 45 | BAB41673.1 | hypothetical protein, similar to signal peptidase II homologue | YaaT | 29.04 | 71.09 | 23.9 | 5.3 |
| 46 | BAB43460.1 | hypothetical protein, similar to TpgX protein | SA2158 | 27.40 | 66.18 | 22.8 | 6.2 |
| 47 | BAB41648.1 | cysteine synthase homologue | cysM | 27.39 | 58.8 | 32.9 | 5.9 |
| 48 | BAB41794.1 | alcohol dehydrogenase I | adh1 | 20.54 | 66.96 | 36.0 | 5.7 |
| 49 | BAB42605.1 | hypothetical protein, similar to tripeptidase | SA1343 | 20.02 | 44.56 | 40.2 | 5.0 |
| 50 | BAB42068.1 | hypothetical protein, similar to 5-oxo-1,2,5-tricarboxilic-3-penten acid decarboxylase | SA0829 | 16.71 | 55.43 | 33.1 | 5.0 |
| 51 | BAB41475.1 | two-component response regulator | lytR | 14.42 | 50.41 | 28.2 | 6.0 |
| 52 | BAB41924.1 | lipoprotein, similar to ferrichrome ABC transporter | CeuA | 11.21 | 71.35 | 37.8 | 9.3 |
| 53 | BAB41404.1 | PTS enzyme II (EC 2.7.1.69), glucose-specific, factor IIA homologue | glcA | 10.49 | 31.42 | 73.9 | 6.4 |
| 54 | BAB42710.1 | alanyl-tRNA synthetase | alaS | 7.41 | 59.93 | 98.5 | 5.1 |
|  |  |  |  |  |  | *(Continued)* | |
| **NO.** | **Accession No** | **Protein name** | **Gene name** | **Score** | **Coverage** | **MW [kDa]** | **calc. pI** |
| 55 | BAB42113.1 | conserved hypothetical protein | SA0872 | 7.33 | 50 | 29.2 | 6.5 |
| 56 | BAB41911.1 | hypothetical protein, similar to choline transporter | SA0678 | 5.85 | 39.48 | 56.0 | 9.4 |
| 57 | BAB42527.1 | hypothetical protein, similar to streptococcal adhesin emb | ebhA | 5.59 | 44.57 | 721.9 | 5.7 |
| 58 | BAB43753.1 | conserved hypothetical protein | SA2448 | 4.93 | 65.22 | 25.9 | 5.2 |
| 59 | BAB41731.1 | RNA polymerase beta chain | rpoB | 2.39 | 55.03 | 133.1 | 5.0 |
| 60 | BAB41712.1 | hypothetical protein, similar to creatine kinase | SA0482 | 2.32 | 59.4 | 38.6 | 5.2 |
| 61 | BAB41931.1 | aminotripeptidase | pepT | 2.31 | 27.7 | 45.8 | 4.9 |
| 62 | BAB42390.1 | DNA mismatch repair protein | mutS | 2.13 | 50.69 | 99.8 | 5.0 |
| 63 | BAB42969.1 | hypothetical protein, similar to transporter | SA1699 | 2.06 | 20.25 | 45.6 | 8.8 |
| 64 | BAB41691.1 | transcription-repair coupling factor | mfd | 1.96 | 50.17 | 134.2 | 5.2 |
| 65 | BAB41239.1 | hypothetical protein, similar to 5'-nucleotidase | SA0022 | 1.91 | 45.85 | 83.4 | 9.2 |
| 66 | BAB42735.1 | hypothetical protein, similar to chorismate mutase | SA1469 | 1.73 | 58.55 | 17.5 | 5.7 |
| 67 | BAB42042.1 | probable cytosol aminopeptidase | ampA | 1.73 | 52.55 | 54.0 | 6.1 |
| 68 | BAB43294.1 | hyaluronate lyase precursor | hysA | 1.68 | 54.39 | 91.8 | 8.1 |
