# Supplementary material for: β-Lactam Antibiotics Enhance the Pathogenicity of Methicillin-Resistant Staphylococcus aureus via SarA-Controlled Lipoprotein-Like Cluster Expression
Source: mBio. 2019 Jun 11;10(3):e00880-19. doi: 10.1128/mBio.00880-19 (PMC6561022; doi:10.1128/mBio.00880-19)
Supplement: TABLE S4 [file mBio.00880-19-st004.docx]

**TABLE S4** Strains and plasmids used in this study.

| **Strain/plasmid** | **Description** | **Reference** |
| --- | --- | --- |
| **Strains** |  |  |
| ***E.coli*** |  |  |
| DH5α | Laboratory strain | Tiangen |
| BL21(DE3) | Protein expression | Tiangen |
| ***S. aureus*** |  |  |
| RN4220 | Restriction deﬁcient cloning host | (1) |
| N315 | USA100 |  |
| MRSA ST88 | Clinical strains | (2) |
| MRSA ST239 |  |  |
| MRSA ST59 |  |  |
| MRSA ST1 |  |  |
| MRSA ST398 |  |  |
| MRSA ST8 | USA300_FPR3757 |  |
| N315Δ*lpl* (USA300Δ*lpl*) | Markerless deletion of  *sa2275*- *sa2273* (*SAUSA300_2430-* *28*) | This study |
| N315Δ*sarA* | Markerless deletion of *sarA* | This study |
| N315Δ*lpl*/pLI-*lpl* (USA300Δ*lpl*/pLI-*lpl*) | *lpl* overexpressed with pLI50 | This study |
| N315Δ*lpl*/pIL50 (USA300Δ*lpl*/pLI50) | Empty PLI50 tranformed strain | This study |
| N315Δ*sarA/*pLI-*sarA* | *sarA* overexpressed with pLI50 | This study |
| N315Δ*sarA*/pIL50 | Empty PLI50 tranformed strain | This study |
| N315/pGFP | pGFG plasmid tranformed N315 strain | This study |
| N315Δ*lpl*/pGFP | pGFG plasmid tranformed N315Δ*lpl* strain | This study |
| N315Δ*lpl*/pXR-*sa2275*-his | pXR-*sa2275*-his plasmid tranformed N315Δ*lpl* strain | This study |
|  |  | *(Continued)* |
| **Strain/plasmid** | **Description** | **Reference** |
| **plasmid** |  |  |
| pGFP | gfp expression with the promoter of S10 ribosomal gene, Amp^R^ Cm^R^ | (3) |
| pET28a-*sa2275* | P*_T7_*-dircted synthesis of *S. aureus* N315 *sa2275* protein fused to an N-terminal his6 tag | This work |
| pET28a-*sarA* | P*_T7_*-dircted synthesis of *S. aureus* N315 *sarA* protein fused to an N-terminal his6 tag | This work |
| pYT3 | *S. aureus*-*E. coli* shuttle vector, temperature sensitive, Amp^R^ in *E. coli* and Tet^R^ in *S. aureus* | (4) |
| pBT2 | *S. aureus*-*E. coli* shuttle vector, temperature sensitive, Amp^R^ in *E. coli* and Cm^R^ in *S. aureus* | (1) |
| pYT3Δ*lpl* | pYT3 derivative, for *sa2275-sa2273* deletion in N315 | This study |
| pBT2Δ*lpl* | pBT2 derivative, for *SAUSA300_2430-* *28* deletion in USA300 |  |
| pYT3Δ*sarA* | pYT3 derivative, for *sarA* deletion in N315 | This study |
| pBT2Δ*agrA* | pBT2 derivative, for *agrA* deletion in N315 and USA300 | This study |
| PLI50 | *S. aureus*-*E. coli* shuttle overexpressed vector, Amp^R^ in *E. coli* and Cm^R^ in *S. aureus* | (5) |
| PLI-*lpl* | pLI50 with *lpl* operon (*sa2275* through *sa227*3 genes) under the control of its endogenous promoter | This study |
| PLI-*sarA* | pLI-*sarA* with a 1×FLAG sequence before the termination codon, under the control of its endogenous promoter | This study |
| pXR | Xylose inducible expression vector, pLI50 with *xylR* and *xylAB* promoter sequence | (6) |
| pXR-*sa2275*-his | pXR containing *sa2275*gene fused to an C-terminal his6 tag | This study |

**REFERENCES**

1. Peng H, Hu Q, Shang W, Yuan J, Zhang X, Liu H, Zheng Y, Hu Z, Yang Y, Tan L, Li S, Hu X, Li M, Rao X. 2017. WalK(S221P), a naturally occurring mutation, confers vancomycin resistance in VISA strain XN108. J Antimicrob Chemother 72:1006-13.

2. Cheng H, Yuan W, Zeng F, Hu Q, Shang W, Tang D, Xue W, Fu J, Liu J, Liu N, Zhu J, Yang J, Hu Z, Yuan J, Zhang X, Li S, Chen Z, Hu X, Rao X. 2013. Molecular and phenotypic evidence for the spread of three major methicillin-resistant *Staphylococcus aureus* clones associated with two characteristic antimicrobial resistance profiles in China. J Antimicrob Chemother 68:2453-7.

3. Ma R, Qiu S, Jiang Q, Sun H, Xue T, Cai G, Sun B. 2017. AI-2 quorum sensing negatively regulates rbf expression and biofilm formation in *Staphylococcus aureus*. Int J Med Microbiol 307:257-67.

4. Yuan W, Hu Q, Cheng H, Shang W, Liu N, Hua Z, Zhu J, Hu Z, Yuan J, Zhang X, Li S, Chen Z, Hu X, Fu J, Rao X. 2013. Cell wall thickening is associated with adaptive resistance to amikacin in methicillin-resistant *Staphylococcus aureus* clinical isolates. J Antimicrob Chemother 68:1089-96.

5. You Y, Xue T, Cao L, Zhao L, Sun H, Sun B. 2014. *Staphylococcus aureus* glucose-induced biofilm accessory proteins, GbaAB, influence biofilm formation in a PIA-dependent manner. Int J Med Microbiol 304:603-12.

6. Wieland KP, Wieland B, Gotz F. 1995. A promoter-screening plasmid and xylose-inducible, glucose-repressible expression vectors for *Staphylococcus carnosus*. Gene 158:91-6.
