# Supplementary material for: β-Lactam Antibiotics Enhance the Pathogenicity of Methicillin-Resistant Staphylococcus aureus via SarA-Controlled Lipoprotein-Like Cluster Expression
Source: mBio. 2019 Jun 11;10(3):e00880-19. doi: 10.1128/mBio.00880-19 (PMC6561022; doi:10.1128/mBio.00880-19)
Supplement: TABLE S5 [file mBio.00880-19-st005.docx]

**TABLE S5** The distribution of *lpl* cluster in major MRSA clones.

| **ST** | **Strains** | **Conserved SarA binding box** | **Lpl locus** | | |
| --- | --- | --- | --- | --- | --- |
| ST5 | N315 | agg**atttaat**cataaaat | SA2275 | SA2274 | SA2273 |
|  | MU50 |  | SAV2487 | SAV2486 | SAV2485 |
|  | MU3 |  | SAHV_2471 | SAHV_2470 | SAHV_2469 |
| ST8 | USA300 |  | SAUSA300_2430 | SAUSA300_2429 | SAUSA300_2428 |
|  | JE2 |  | B7H15_13840 | **-** | B7H15_13835 |
| ST105 | JH1 |  | SaurJH1_2566 | SaurJH1_2565 | SaurJH1_2564 |
|  | JH9 |  | SaurJH9_2514 | SaurJH9_2513 | SaurJH9_2512 |
| ST228 | 18583 |  | SAI8T7_1018490 | SAI8T7_1018480 | SAI8T7_1018470 |
|  | 18341 |  | SAI6T6_1018450 | SAI6T6_1018440 | SAI6T6_1018430 |
|  | 16125 |  | SAI5S5_1018450 | SAI5S5_1018440 | SAI5S5_1018430 |
|  | 15532 |  | SAI3T3_1018510 | SAI3T3_1018500 | SAI3T3_1018490 |
|  | 10497 |  | SAI2T2_1018520 | SAI2T2_1018510 | SAI2T2_1018500 |
|  | 10388 |  | SAI1T1_2018510 | SAI1T1_2018500 | SAI1T1_2018490 |
|  | 16035 |  | SAI4T8_1018520 | SAI4T8_1018510 | SAI4T8_1018500 |
|  | 18412 |  | SAI7S6_1018510 | SAI7S6_1018500 | SAI7S6_1018490 |
| ST239 | XN108 |  | SAXN108_2735 | SAXN108_2734 | **-** |
|  | TW20 |  | SATW20_26100 | SATW20_26090 | **-** |
