# Supplementary material for: β-Lactam Antibiotics Enhance the Pathogenicity of Methicillin-Resistant Staphylococcus aureus via SarA-Controlled Lipoprotein-Like Cluster Expression
Source: mBio. 2019 Jun 11;10(3):e00880-19. doi: 10.1128/mBio.00880-19 (PMC6561022; doi:10.1128/mBio.00880-19)
Supplement: TABLE S6 [file mBio.00880-19-st006.docx]

**TABLE S6** Primers used in this study.

| **Name** | **Sequence (5′→3′)** | **Product Size (bp)** |
| --- | --- | --- |
| 5F | gtattattaatattggaatttgaac | 2735 |
| 4F | caattgaaaaacgatgattac | 1219 |
| 3F | taacgctcggtatatgtttgg | 750 |
| 3R | cagtaggtttgaaattaataccatc |  |
| RT-*sa2273* | ttagttttgttcatgattcat |  |
| up_*lpl* (Hind III ) fwd | ccc**aagctt**agaatgtaatgaaagaatgat | 898 |
| up_*lpl* (Sal I ) rev | aaa**gtcgac**ttcacatccccatttttattt |  |
| down_*lpl* (Sal I ) fwd | agc**gtcgac**atatcaaataaattctgacaa | 873 |
| down_*lpl* (BamH I ) rev | acg**ggatcc**actttatgtaatctatgcctc |  |
| up_*sarA* (Hind III ) fwd | ggg**aagctt**tagaaaagcgttgatttgggt | 869 |
| up_*sarA* (Sal I ) rev | cgc**gtcgac**gtttaaaacctccctatttga |  |
| down_*sarA* (Sal I ) fwd | agc**gtcgac**ttttgtttagcgcaatttggt | 862 |
| down_*sarA* (BamH I ) rev | cgc**ggatcc**cttctaaaagtgaatcatacg |  |
| up_*agrA* (BamH I ) fwd | cgc**ggatcc**ctacaaatacaagttcaaac | 836 |
| up_ *agrA* (Sal I ) rev | gatttacaattgaatacgccgacattcacatccttatggctag |  |
| down_ *agrA* (Sal I ) fwd | ctagccataaggatgtgaatgtcggcgtattcaattgtaaatc | 827 |
| down_ *agrA* (Hind III) rev | ggg**aagctt**tatgggataacgctgaagat |  |
| c_*lpl* fwd | ttttcaagacaaatagcacctgga | 4748 |
| c_*lpl* rev | attgttgttgggaggaagagagtg |  |
| pLI*-lpl* (BamH I ) fwd | aaa**ggatcc**taaaatgccgaatggttcaaa | 3094 |
| pLI-*lpl* (Hind III ) rev | ccc**aagctt**ttagttttgttcatgattcat |  |
| PLI-*sarA* (BamH I ) fwd | aaa**ggatcc**ggccagattctaatgggcata | 1451 |
| PLI-*sarA* (Hind III ) rev | ccc**aagctt**ttacttatcgtcgtcatccttgtaatctagttcaatttcgttgtttgc |  |
| pET28*-sa2275*-his (-sp) (BamH I ) fwd | aac**ggatcc**tgcggaatgaaaaaggaag | 708 |
| pET28-*sa2275*-his (-sp) (Sal I ) rev | agc**gtcgac**ctattcagtaggtttgaaatt |  |
| pET28-*sarA*-his (BamH I ) fwd | cgc**ggatcc**atggcaattacaaaaatcaat | 375 |
| pET28*-sarA*-his (Sal I ) rev | cgc**gtcgac**ttatagttcaatttcgttgtt |  |
| EMSA-*lpl*^P^ fwd | ccacgaggatttaatcataaaatttcctctagtattattaatattggaatttgaac | 56 |
| EMSA-lpl^P^ rev | gttcaaattccaatattaataatactagaggaaattttatgattaaatcctcgtgg |  |
| EMSA-*lpl*^PM^ fwd | ccacgagggggggggcgggggggttcctctagtattattaatattggaatttgaac | 56 |
| EMSA-*lpl*^PM^ rev | gttcaaattccaatattaataatactagaggaacccccccgccccccccctcgtgg |  |
|  | *(Continued)* | |
|  |  |  |
| **Name** | **Sequence (5′→3′)** | **Product Size (bp)** |
| *xylR-xylAP* (EcoR I ) fwd | ccg**gaattc**aagcttagcaactt | 1687 |
| *xylR-xylAP* (Kpn I ) rev | cgg**ggtacc**aatattcctcctacattttag |  |
| pXR*-sa2275*-his (his6) fwd | ctaaaatgtaggaggaatattatgatgattcattcaagaaag | 804 |
| pXR*-sa2275*-his (his6) rev | agcgtcgacctaatgatgatgatgatgatgttcagtaggtttgaaattaatac |  |
| *sa2275* fwd  *sa2275* rev | gttgaggctttggctgtatttagta | 182 |
|  | caaactcttcatctcgaaaaccttc |  |
| *sa2274* fwd | ttggattgttgattctgagatggta | 147 |
| *sa2274* rev | gcataccctttactatcttcccaca |  |
| *sa2273* fwd | aaataccctgtcaagatggaacg | 190 |
| *sa2273* rev | attgatactttgcggaataacttg |  |
| 16S *rRNA* fwd | gctcgtgtcgtgagatgttgg | 195 |
| 16S *rRNA* rev | tttcgctgccctttgtattgt |  |
